# Transcriptomic comparison between *Brassica oleracea* and rice (*Oryza sativa*) reveals diverse modulations on cell death in response to *Sclerotinia sclerotiorum*

**DOI:** 10.1038/srep33706

**Published:** 2016-09-20

**Authors:** Jiaqin Mei, Yijuan Ding, Yuehua Li, Chaobo Tong, Hai Du, Yang Yu, Huafan Wan, Qing Xiong, Jingyin Yu, Shengyi Liu, Jiana Li, Wei Qian

**Affiliations:** 1 College of Agronomy and Biotechnology, Southwest University, Chongqing 400716, China; 2 Key Laboratory of Biology and Genetic Improvement of Oil Crops, Ministry of Agriculture, Oil Crops Research Institute of the Chinese Academy of Agricultural Sciences, Wuhan 430062, P.R. China; 3 College of Plant Protection, Southwest University, Chongqing 400716, China; 4 School of Computer and Information Science, Southwest University, Chongqing 400716, China

## Abstract

*Sclerotinia* stem rot caused by *Sclerotinia sclerotiorum* is a devastating disease of *Brassica* crops, but not in rice. The leaves of a rice line, a partial resistant (R) and a susceptible (S) *Brassica oleracea* pool that bulked from a resistance-segregating F_2_ population were employed for transcriptome sequencing before and after inoculation by *S. sclerotiorum* for 6 and 12 h. Distinct transcriptome profiles were revealed between *B. oleracea* and rice in response to *S. sclerotiorum*. Enrichment analyses of GO and KEGG indicated an enhancement of antioxidant activity in the R *B. oleracea* and rice, and histochemical staining exhibited obvious lighter reactive oxygen species (ROS) accumulation and cell death in rice and the R *B. oleracea* as compared to that in the S *B. oleracea*. Significant enhancement of Ca^2+^ signalling, a positive regulator of ROS and cell death, were detected in S *B. oleracea* after inoculation, while it was significantly repressed in the R *B. oleracea* group. Obvious difference was detected between two *B. oleracea* groups for WRKY transcription factors, particularly for those regulating cell death. These findings suggest diverse modulations on cell death in host in response to *S. sclerotiorum*. Our study provides useful insight into the resistant mechanism to *S. sclerotiorum.*

*Sclerotinia sclerotiorum* is a necrotizing fungal pathogen that infects hundreds of plant species, including important crops such as soybean and rapeseed (*Brassica napus* L.), but it does not infect rice (*Oryza sativa*)^1^,^2^. Many studies have been performed to investigate the defense response and molecular mechanisms in *Brassica* hosts against *S. sclerotiorum*. Several quantitative trait loci (QTL) for *S. sclerotiorum* resistance have been mapped in rapeseed and its relatives^3^,^4^,^5^,^6^,^7^. A number of enzymes were revealed to be associated with resistance to *S. sclerotiorum*, such as polygalacturonase-inhibiting protein, germin-like proteins, several mitogen-activated protein kinases (MAPKs), and WRKY transcription factors (TFs)^8^,^9^,^10^,^11^,^12^,^13^. Transcriptional studies in rapeseed revealed the biological alterations in response to *S. sclerotiorum*, including oxidative burst, cell wall enforcement or modification, secondary metabolism, calcium binding, antioxindation, glutathiones (GSHs) metabolism, glucosinolates metabolism, biosynthesis of lignins, TFs, carbohydrate metabolism, and energy metabolism^4^,^11^,^12^,^14^,^15^,^16^,^17^,^18^,^19^. Due to the lack of high resistance source, however, the molecular mechanism of resistance against *S. sclerotiorum* was still poorly understood.

Recently, a wild *B. oleracea* genotype, ‘C01’, has been identified to be with partial resistance against *S. sclerotiorum*, exhibiting significant higher resistance level than that of a partial resistant *B. napus* variety ‘Zhongshuang 9’^20^. The resistance QTL have been identified from an F_2_ population derived from ‘C01’^3^ and it has been proved that ‘C01’ was effective in improving *Sclerotinia* resistance of *Brassica* species^21^,^22^. In order to investigate the response to *S. sclerotiorum* in hosts with different resistance levels, rice with nonhost resistance to *S. sclerotiorum*, and a partial resistant (R) and a susceptible (S) pool that bulked from the extreme resistant and susceptible genotypes in the F_2_ population derived from ‘C01’ were compared for gene expression alterations in leaf before and after inoculation. Significant differences were found for antioxidation, Ca^2+^ signalling, WRKY TFs and ribosome which are in association with modulating cell death of host in defense to pathogens. Our study provides new insights into the resistant mechanism to *S. sclerotiorum*.

## Results

### Resistance performance and transcriptome sequencing

**Figure 1:**
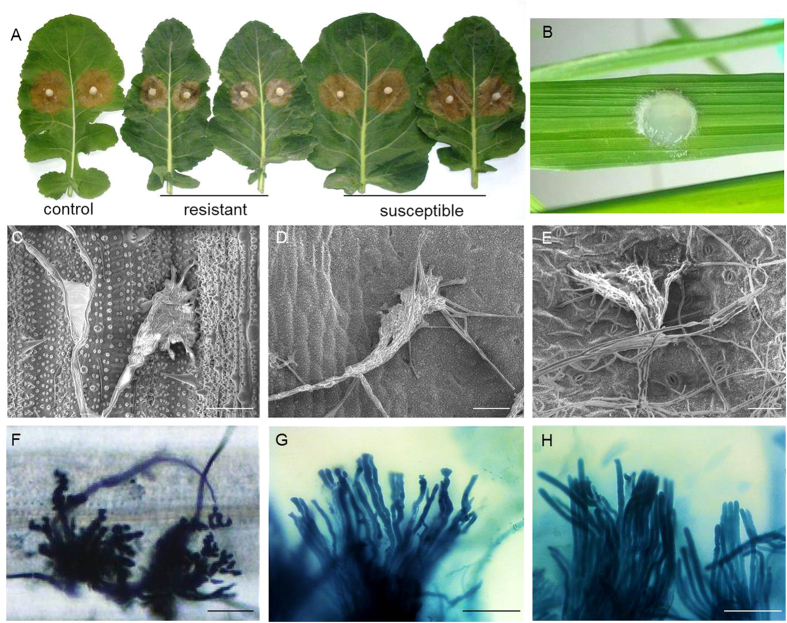
Performances of *Sclerotinia sclerotiorum*-infected leaves of *Brassica oleracea* and rice. (**A**) Symptoms of resistant and susceptible plants of *Brassica oleracea* at 3 days after inoculation by *S. sclerotiorum.* The left side exhibits the performance of control ‘Zhongshuang 9’ which is a partial resistant rapeseed variety against *S. sclerotiorum*. (**B**) Performance of rice at 3 days after inoculation by *S. sclerotiorum*. (**C–E**) Infection cushions on leaves of rice, the partial resistant and the susceptible *B. oleracea* at 12 hpi, respectively (observed using electron microscope). (**F–H**) Infection cushion on rice leaf and invasive hyphae in leaves of the partial resistant and the susceptible *B. oleracea* at 36 hpi, respectively. Scale bar = 50 μm.

Different phenotypic responses were observed among rice, the R and the S *B. oleracea* group in response to *S. sclerotiorum* (Fig. 1). No symptom was visible on the inoculated rice leaf at 3 days after inoculation (dai), while the lesion size of the R and the S *B. oleracea* group averaged on 5.4 cm^2^ (4.5~6.2 cm^2^) and 9.8 cm^2^ (8.5~12.3 cm^2^) at 3 dai, respectively. Though infection cushions could be found from the inoculated leaves of rice, the partial resistant and the susceptible *B. oleracea* at 12 hours post-inoculation (hpi), it seems that the infection cushions on susceptible *B. oleracea* growed faster (already formed many hyphae) and stronger (in a bigger shape and with more ‘fingers’ which will turn into invasive hyphae soon) than that on rice and the resistant line. At 36 hpi, infection cushions were still the leading structures on the leaf surface of rice, while benched and top-aberrant hyphae were dominant at the lesion edge of the partial resistant *B. oleracea*, differing from the thick and fluent hyphae in the susceptible *B. oleracea*. These observations indicate that the propagation of *S. sclerotiorum* was hindered entirely in rice and partially in the R *B. oleracea*.

Each of three libraries of rice (constructed at 0, 6 and 12 hpi, named as Os0, Os6 and Os12, respectively), the R (R0, R6 and R12) and the S (S0, S6 and S12) *B. oleracea* group were sequenced on an Illumina Hiseq 2000^TM^ platform, yielding more than 25 million clean reads for each *B. oleracea* sample and over 15 million for rice. Of these, 72.3% and 76.8% on average were aligned to the reference genome of *B. oleracea* and rice, respectively (Supplementary Table 1). A total of 34,550 and 27,405 unigenes were detected from *B. oleracea* and rice, of which 7,230 *B. oleracea* genes (4,961 from R group, 4,890 from S group, with 2,621 overlap genes) and 3,470 rice genes exhibited more than two-fold transcriptional differences between neighboring time points. The real-time qRT-PCR (qRT-PCR) analysis for 46 differentially expressed genes (DEGs) from *B. oleracea* and five DEGs of interest showed general agreement with the RNA-seq data (Supplementary Figure 1).

### Comparison of gene expression alterations after inoculation between *B. oleracea* and rice

**Figure 2:**
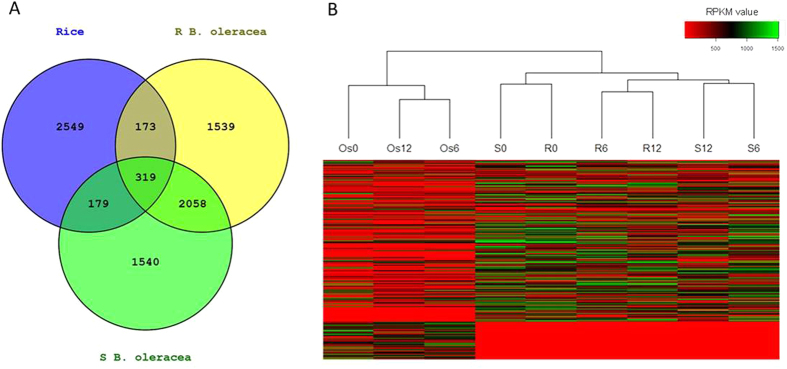
DEGs in rice and *Brassica oleracea* in response to *Sclerotinia sclerotiorum.* (**A**) *Venn* diagram of DEGs in rice and *B. oleracea* during whole infection. (**B**) Heatmap of inoculated and non-inoculated *B. oleracea* and rice according to the RPKM values of DEGs. Os, rice; R, Resistant *B. oleracea*; S, susceptible *B. oleracea*; Os0, R0 and S0, non-inoculated controls; Os6, R6 and S6, samples collected 6 hpi; Os12, R12 and S12, samples collected 12 hpi.

**Figure 3:**
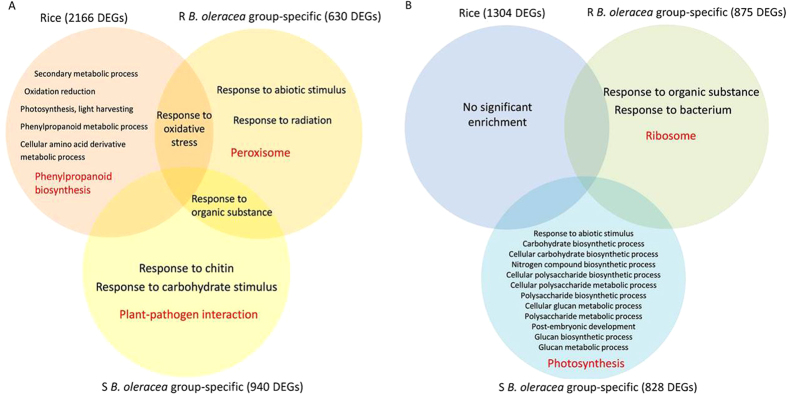
Significantly enriched GO (in black) and KEGG (in red) terms among up-regulated (**A**) and down-regulated DEGs (**B**) in rice, resistance (R) and susceptible (S) *Brassica oleracea*.

After aligning all DEGs to *Arabidopsis* homologous genes, 671 overlapped genes were found in rice and *B. oleracea* (Fig. 2A, Supplementary Table 2). These genes significantly enriched in Gene Ontology (GO) terms related to response to abiotic stimuli, such as response to light stimulus, chemical stimulus and stress (Supplementary Figure 2). However, an obvious difference for transcriptome profile was revealed between *B. oleracea* and rice by the heatmap upon all DEGs (Fig. 2B). In order to get insight into the resistance mechanism that overlapped in rice and resistant *B. oleracea*, GO analysis was conducted separately on the up- and down-regulated DEGs, and in two *B. oleracea* groups on the up- and down-regulated DGEs that specifically found in each group (Fig. 3, Supplementary Table 3).

No significant biology process was enriched in rice by analyzing 1,304 down-regulated DEGs, while 2,166 up-regulated DEGs significantly enriched in ‘response to oxidative stress’, ‘secondary metabolic process’, ‘oxidation reduction’, ‘photosynthesis, light harvesting’, ‘phenylpropanoid metabolic process’ and ‘cellular amino acid derivative metabolic process’. Among these, the term ‘response to oxidative stress’ was also significantly enriched in the R *B. oleracea* group by analyzing 630 up-regulated DEGs that were R-group specific. No common biology process was detected between rice and the S *B. oleracea* group (Fig. 3).

In details, a total of 26 DEGs were involved in the term of ‘response to oxidative stress’ in rice (*FDR* = 3.91e^−4^), including 25 peroxidases (Supplementary Table 4). These antioxidant genes were sharply induced at 6 hpi, and 16 of these were maintained at high expression levels at 12 hpi. For example, a precursor of cationic peroxidase SPC4 (LOC_Os01g73200) was up regulated for 390 folds at 6 hpi and even increased for 420 folds at 12 hpi. Being similarly, the GO term ‘response to oxidative stress’ that significantly enriched in the R *B. oleracea* group (*FDR* = 1.01e^−02^) involves 19 antioxidant genes, including 9 peroxidases, 7 catalases (CATs) and 3 superoxide dismutases (SODs). These genes were increased for abundance by up to 45 folds in the R group at 6 hpi. These imply a notable inducement of antioxidant activity in rice and the resistant *B. oleracea* after inoculation.

**Figure 4:**
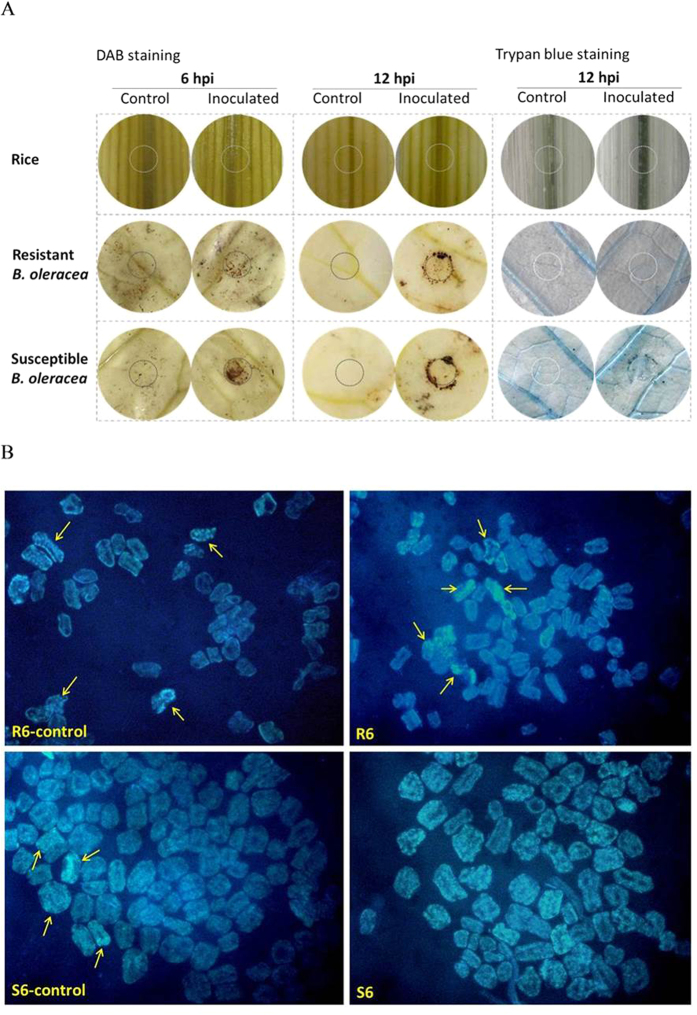
H_2_O_2_ accumulation and cell death in rice and *Brassica oleracea* after inoculation with *Sclerotinia sclerotiorum*. Controls (inoculated with PDA agar discs) and infected leaves were stained with 3,0-diaminobenzidine (DAB) to visualize the H_2_O_2_ accumulation (**A**), with trypan blue for necrosis (**A**), and with DAPI for apoptosis (**B**), respectively. Open cycle on each leaf in A indicates the inoculation site. Arrows in B indicates typical apoptosis cells (all cells of S6 are apoptosis, thus are not indicated).

**Figure 5:**
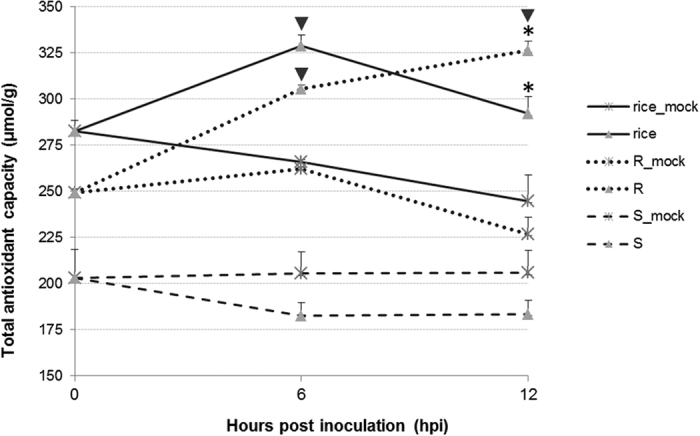
The total antioxidant capacity of rice and resistant (R) and susceptible (S) *Brassica oleracea* after inoculation with *Sclerotinia sclerotiorum*. Mocks were inoculated with agar discs. Asterisks indicate significant difference of inoculated samples with corresponding mocks at a same time point, and inverted triangles represent significant difference of samples at a certain time point comparing to a former time point (*P* < 0.01).

The high antioxidant activity was also observed in rice and the resistant *B. oleracea* by histochemical staining study and enzyme activity study. H_2_O_2_ is one of the prominent reactive oxygen species (ROS) which cause cell death in host^23^. By staining leaves with 3,0-diaminobenzidine (DAB) which visualizes the accumulation of H_2_O_2_, we found obvious stained dots at both 6 and 12 hpi in the susceptible *B. oleracea*, but no stained dot in rice during the whole infection and only a few stained dots on the edge of inoculation point in the resistant *B. oleracea* at 12 hpi (Fig. 4). This result was supported by the total antioxidant capacity assay, which revealed singificant enhanced antioxidant enzyme activity in rice (6 hpi) and R *B. oleracea* (both 6 and 12 hpi) after inoculation (*P* < 0.01) (Fig. 5). Correspondingly, the trypan blue staining revealed heavier necrosis in the susceptible *B. oleracea* than that in the resistant *B. oleracea* and rice at 12 hpi, and the DAPI staining presented obvious higher ratio of apoptosis in the susceptible *B. oleracea* than that in the resistant *B. oleracea* at 6 hpi (Fig. 4).

### Transcriptome alterations during inoculation within *B. oleracea*

The heatmap of DEGs revealed similar transcriptome pattern between the R and S *B. oleracea* groups prior to inoculation (Fig. 2), suggesting a high similarity in their genetic background. However, distinct expression profiles were exhibited between the two groups after inoculation (Fig. 2), reflecting different responses to *S. sclerotiorum* between resistant and susceptible *B. oleracea*.

Kyoto Encyclopedia of Genes and Genomes (KEGG) analysis showed that the term ‘ribosome’ was significantly enriched among 875 down-regulated DEGs specific to the R group (*P*_*Bonferroni*_ = 6.69e^−21^), while the term ‘Photosynthesis’ was significantly enriched among 828 down-regulated DEGs specific to the S group (*P*_*Bonferroni*_ = 1.13e^−03^) (Fig. 3, Supplementary Table 3). It suggests a fast repression on translation in the resistant *B. oleracea*, and a significant photosynthetic damage in the susceptible *B. oleracea* during infection.

By analyzing the R-group-specific up-regulated DEGs, significant enrichment was detected on ‘Peroxisome’ (*P*_*Bonferroni*_ = 2.99e^−04^), which mainly performs antioxidant function in response to stress^24^. These DEGs involved in ‘Peroxisome’ were the same as those in the GO term ‘response to oxidative stress’ in the R group which has been mentioned above. The term of ‘Plant-pathogen interaction’ was significantly enriched by the S-group-specific up-regulated DEGs (*P*_*Bonferroni*_ = 1.91e^−05^) (Fig. 3, Supplementary Table 3). By deep comparison of DEGs in ‘Plant-pathogen interaction’ between the two groups, we found obvious different alterations on two sub-terms of ‘Plant-pathogen interaction’, i.e., the Ca^2+^ signalling and the WRKY TFs. The details were shown below.

### Ca^2+^ signalling

Among 196 genes that involved in Ca^2+^ signalling in the reference genome of *B. oleracea* (http://www.genome.jp/kegg-bin/show_pathway?ko04626+K13448), 37 exhibited expression changes after infection in the R and S groups (Supplementary Table 4). As compared with 0 hpi, one *CNGC12* (Bol030676) which transports Ca^2+^ from the apoplast into the cell^25^,^26^ was up-regulated in the S group for 14 folds at 6 hpi and additional five folds at 12 hpi, while it was slightly repressed in the R group at 6 hpi and up-regulated only for three folds at 12 hpi. The other 36 DEGs were found to encode Ca^2+^ -sensor proteins, such as calmodulin (CaM), CaM-like proteins (CMLs) and calcium-dependent protein kinases (CDPKs)^27^. Of these, 22 genes were up regulated among 25 DEGs that detected in the S group, while 15 genes were down regulated among 21 DEGs that detected in the R group. Two of these DEGs, *CAM5* (Bol020573) and a *CML37* (Bol035298), were selected and certified for gene expression profile by qRT-PCR (Supplementary Figure 1). These findings indicate that the Ca^2+^ signalling was possibly promoted in the susceptible group but suppressed in the resistant *B. oleracea* after inoculation with *S. sclerotiorum*.

### WRKY TFs

A total of 50 DEGs encoding WRKY TFs were found in *B. oleracea*, including 34 from the S group and 32 from the R group (Supplementary Table 4). More WRKYs were up-regulated in the S group (25 in 28 DEGs at 6 hpi and six in eight DEGs at 12 hpi) than in the R group (11 in 24 DEGs at 6 hpi and seven in ten DEGs at 12 hpi). Of these, only eight up-regulated DEGs overlapped in the two groups, including *WRKY6*, *28*, *30* (2 copies), *33*, *45*, *53* and *75* which have been verified to positively regulate the resistance to *S. sclerotiorum*^11^,^12^,^13^,^15^,^16^,^19^. It suggested diverse activation on WRKY TFs in the resistant and susceptible *B. oleracea*.

Interestingly, converse regulation patterns were observed between the two groups at 6 hpi for *WRKY50* (Bol022304 and Bol012741), *WRKY51* (Bol015902 and Bol019066) and *WRKY70* (Bol044275 and Bol002548) which are positively modulators of the downstream salicylic acid (SA)-mediated signaling^28^,^29^,^30^. Among these, five were down-regulated in the R group, whereas four were significantly up-regulated in the S group (Supplementary Table 4). The expression of two *WRKY50* (Bol022304 and Bol012741), a *WRKY51* (Bol015902), two *WRKY70* (Bol044275 and Bol002548) and other four *WRKY*s (Bol000927, Bol036034, Bol040536 and Bol042130) were certified by qRT-PCR (Supplementary Figure 1). Accordantly, two homologs of *PR-1* (Bol019271 and Bol006477), the defense marker in SA signalling, were repressed specifically in the R group at 6 hpi for 2.3 and 25.2 folds, respectively. These findings seem to indicate a potential repression on SA signalling in the resistant *B. oleracea.*

## Discussion

Many attempts have been made to understand the resistance mechanism of *Brassica* crops against *S. sclerotiorum* and revealed various aspects of defense response to this necrotrophic pathogen^4^,^11^,^12^,^14^,^15^,^16^,^17^,^18^,^19^. We also found great alterations in *B. oleracea* in response to *S. sclerotiorum*, such as plant hormone signal transduction, plant–pathogen interaction, and biosynthesis of glucosinolate, phenylpropanoid, flavonoid and other secondary metabolites (Supplementary Table 5). However, when comparing the hosts with various level of resistance, we found obvious difference for antioxidant activity between the resistant (rice and the R *B. oleracea*) and the susceptible host (the S *B. oleracea*) at the early infection phase by *S. sclerotiorum*.

ROS play a crucial role in defense reaction against pathogens but also cause damages and cell death in host^23^. In previous studies, several antioxidation-related proteins or genes have been revealed to be associated to the defence to *S. sclerotiorum* in *B. napus*, such as GSTU11, GSTU12, SOD, and peroxidase^4^,^15^,^17^,^18^,^31^. An enzyme assay indicated a slight higher activity of antioxidant enzymes in resistant *B. napus* genotype than that in the susceptible genotype^18^. In our study, dozens of genes encoding ROS scavenging enzymes such as peroxidase, SOD and CAT were strongly increased in rice and the R *B. oleracea*. In addition, eight glutathione transferases (GSTs) which positively regulate GSH content and reduce ROS^32^ were up-regulated specifically in the R group (Supplementary Table 4). The histochemical staining and the antioxidant capacity assay further proved stronger antioxidant activity and lighter cell death in rice and the R *B. oleracea* than that in the susceptible *B. oleracea*. Therefore, we reason that the effective ROS scavenging systems in the resistant hosts possibly help to alleviate and delay the cell death, and thus defend the invasion of the necrotrophic fungus which obtains nutrients from necrotic host tissues^33^,^34^.

By further comparison within *B. oleracea*, we detected an enhanced Ca^2+^ signalling in the S *B. oleracea* group according to the great up-regulation of *CNGC12*, *CaM*s and *CDPKs*. In a previous report^15^, several genes involved in calcium signal transduction were found to be up-regulated in a susceptible *B. napus* variety, including one gene (AT3G50770) which is homologous to a calmodulin-related gene (Bol002305) induced specifically in the S group in our study. Conversely, though the *CNGC12* (Bol030676) was slightly enhanced at 12 hpi in the R group, the *CaM*s and *CDPK*s were repressed in the R group. Transient elevation of cytosolic Ca^2+^ always occurs after pathogen perception to activate downstream signalling cascade which includes activation of CaM and protein kinases, and generation of nitric oxide (NO) and ROS^35^,^36^,^37^,^38^,^39^. The activation of CDPKs, generation of NO and ROS, and increase in Ca^2+^ binding have been demonstrated to positively regulate programmed cell death (PCD)^40^. In multicellular organisms, apoptosis and autophagy are recognized as two principal means by which these genetically determined cell deaths occur. It was found that *S. sclerotiorum* hijacks host pathways via oxalic acid (OA) and induces cell death in host plant tissue resulting in hallmark apoptotic features in a time and dose dependent manner, while the autophagy is a defense response of plant in *S. slerotiorum*-plant interaction^41^. In the present study, heavier apoptosis was caused by *S. sclerotiorum* in susceptible *B. oleracea* as compared with that in resistant *B. oleracea*. Therefore, the enhanced Ca^2+^ signalling in the S group possibly promotes apoptosis and thus helps the propagation of *S. sclerotiorum*, while the suppressed Ca^2+^ signalling in the R group may contribute to the inhibition the expansion of nectrophic hyphae.

In this study, diverse WRKY TFs were activated in the resistant and susceptible *B. oleracea*. Except for a few WRKYs that activated in both groups, we further found specific changes for some WRKYs between the two groups. For example, we found up-regulation of *WRKY4* (Bol010361) specifically in the R group (Supplement Table 4). It is reported that *WRKY4* in *Tamarix hispida* activated SODs and peroxidase under stress, thus protected cells from death^42^. Whereas, two copies of *WRKY11* (Bol017448, Bol033735) and one *WRKY17* (Bol041910) were up-regulated only in the S group (Supplement Table 4). In previous studies, *WRKY17* from cotton (*Gossypium hirsutum*) increased ROS level and enhanced oxidative damage in transgenic tobacco (*Nicotiana benthamiana*) in response to stresses^43^, and *WRKY11* also positively regulated ROS and caused severe cell death in tobacco leaves^44^. It provides another proof on that, the resistant *B. oleracea* possibly activated antioxidant enzymes to scavenge ROS, while the susceptible *B. oleracea* promoted the accumulation of ROS. In addition, *WRKY50*, *WRKY51* and *WRKY70* were revealed to positively modulate the downstream SA-signalling which regulates the HR based PCD and negatively regulate the resistance against necrotrophic pathogens^28^,^29^,^30^. The unique repression of these *WRKY*s (Bol012741, Bol022304, Bol015902, Bol019066 and Bol044275) and the *PR-1* homologs (Bol019271 and Bol006477) in the R group indicated a repression of SA signalling in resistant *B. oleracea* in response to *S. sclerotiorum*. These data suggest a more effective *WRKY* network in resistant *B. oleracea* in protecting the host cells from death.

Ribosome is the place to biosynthesize proteins in eukaryotes, and the inactivation of ribosome by ribosome-inactivating proteins (RIPs) in plants is association with broad-spectrum resistance to pathogens^45^,^46^,^47^. In this study, a fast and severe repression on genes encoding ribosome proteins was detected in the resistant *B. oleracea* after inoculation. It seems that the RIP-dependent resistance may be also associated with modulation of cell death in host^46^,^47^. Further investigations are needed in future research.

## Conclusions

**Figure 6:**
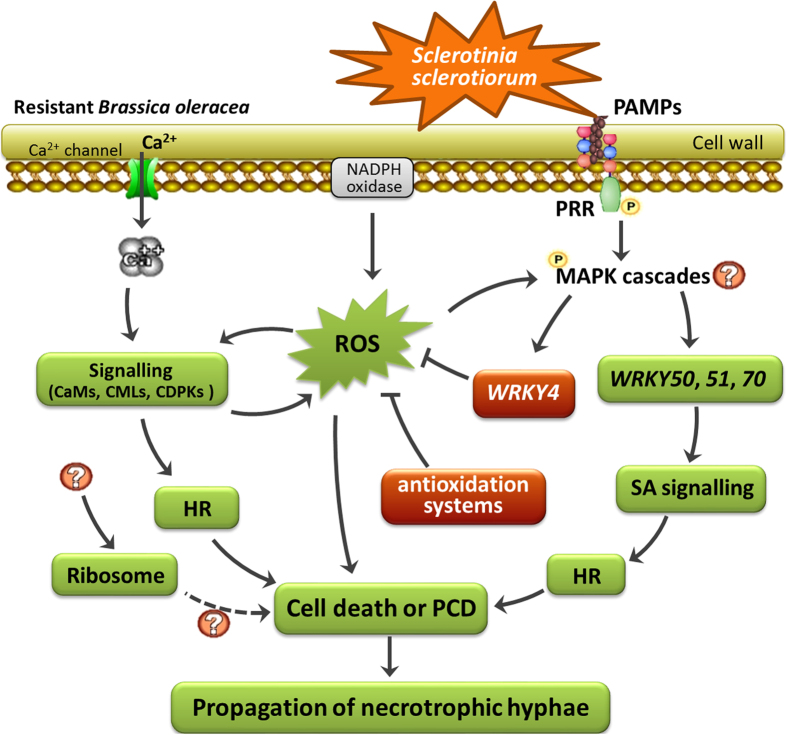
Putative network in resistant *Brassica oleracea* to impair propagation of *S*clerotinia *sclerotiorum* during early infection phase (0 to 12 hpi) via modulating cell death and programmed cell death (PCD). The enhanced and repressed nodes were indicated in red and green backgrounds, respectively.

High antioxidant activity was induced in rice and the resistant *B. oleracea* during the inoculation of *S. sclerotiorum*. The resistant *B. oleracea* possesses an efficient network to impair cell death, including the activation on antioxidant genes and WRKYs, and the suppression on Ca^2+^ signalling and perhaps ribosome (Fig. 6). This study reveals diverse modulations on cell death among hosts, and suggests an important role of impairing cell death via multiple ways in the resistant *B. oleracea* to defend against *S. sclerotiorum.*

## Methods

### Plant materials

The resistance value against *S. sclerotiorum* was previously recorded over two years in an F_2_ population that composed of 149 vegetatively propagated lines derived from a cross between ‘C01’ and a susceptible cultivated genotype ‘C41’^3^,^20^. Based on the resistance performance, three extrem resistant F_2_ lines were selected and pooled with ‘C01’ as the R group, and six highly susceptible F_2_ lines were bulked with ‘C41’ as the S group. The two groups exhibited 94.7% genetic difference within the QTL region (18 of 19 QTL-linked markers exhibited different band patterns in the two groups), and 94.9% genetic similarity in the non-QTL region (410 of 432 markers showed the same band patterns in the two groups). The plants were grown in the field, with 30 cm inter-row and 25 cm intra-row spacing. A rice line (*O. sativa* ssp. *indica*), ‘93–11’ was grown in the field under normal management. All materials were moved into growth chamber one week before inoculation.

### Inoculation and tissue harvest

A *S. sclerotiorum* isolate from a previous study^3^ was used. Mycelium-agar plugs (6-mm-diameter) punched from the growing margin of 3-day-old cultures of *S. sclerotiorum* on potato dextrose agar medium were used as inoculum. The third true leaf at nine-leaf stage of *B. oleracea* and the second leaf of rice seedling were inoculated by attachment of the prepared fungal plugs. The inoculated leaves were sealed with cling film and incubated at 22 °C and 85% relative humidity. As a criterion of resistance reaction, lesion size was measured at 3 dai. The resistance evaluation was performed across two years with three replications. Leaf tissues extending 10 mm beyond the inoculation site at 0, 6, and 12 hpi were excised for RNA extraction.

### Transcriptome sequencing and qRT-PCR analysis

Total RNA was extracted using TRNzol-A + Reagent (TianGen, Beijing, China) and the *B. oleracea* RNA were pooled according to resistance level at each time point, resulting in three R and three S samples, i.e. R0, R6, R12, S0, S6 and S12. Accordingly, the RNA samples of rice were encoded as Os0, Os6 and Os12. Nine library preparations were generated and sequenced on an Illumina Hiseq 2000^TM^ platform. The sequences were base-called and quality-checked by the Illumina data processing pipeline. The raw reads were filtered to obtain high-quality clean reads by removing adaptor sequences, duplicated sequences, reads containing more than 5% “N” (i.e., ambiguous bases in reads), and reads in which more than 50% of the bases showed a *Q*-value (i.e., Bonferroni-adjusted *P* value) ≤ 5. Clean reads were aligned to *B. oleracea* v1.0 genome sequence (http://brassicadb.org/brad/index.php) and the rice genome annotation project data (http://rice.plantbiology.msu.edu/downloads_gad.shtml) using SOAP2, respectively. The uniquely mapped reads were used for gene expression analysis. The abundances of all unigenes were estimated using RPKM (reads per kilobyte per million mapped reads)^48^. DEGs were identified according to Audic and Claverie^49^ and Dang *et al.*^50^. The threshold determining the significance of DEGs among multiple tests was set at a false discovery rate (*FDR*) ≤ 0.001 and |log_2_(folds change)| ≥ 1.

Heatmap was constructed among all samples by the ‘gplots’ package of R using the function ‘heatmap.2.’ . All DEGs of rice and *B. oleracea* were firstly aligned to *Arabidopsis* genes according to information from http://plants.ensembl.org/Oryza_sativa/Info/Index and http://www.ocri-genomics.org/bolbase/at_synviewer.htm, and the expressions of the corresponding *Arabidopsis* homologs were then used to build the heatmap.

Functional enrichment analyses, consisting of GO and KEGG analyses, were performed using the ultra-geometric test^51^. A *FDR*-value ≤ 0.05 was set as the threshold to determine significant enrichments of GO terms and KEGG pathways in *B. oleracea* and rice.

Analysis of qRT-PCR was performed with three replications using a CFX96™ Real-Time PCR Detection System to validate the transcription patterns. The PCR cycling conditions comprised 1 cycle of 95 °C for 30 s, then 39 cycles of 95 °C for 5 s and 55–70 °C for 1 min, followed by a melting curve ramping from 65 °C to 95 °C with temperature increasing by 0.5 °C every 5 s (1 cycle). *Actin3* and *Actin7* were used as the internal control.

### Histochemical staining

To detect hydrogen peroxide *in situ*, inoculated leaves and mocks (inoculated with PDA agar discs) were infiltrated with DAB (Sigma-Aldrich; 1 mg/ml DAB-HCl, pH 3.8) solution at 6 and 12 hpi according to Fryer *et al.*^52^. Trypan blue staining was applied as described by Frye and Innes^53^ to visualize the mycelium of pathogen in host leaves at 36 hpi, and to reveal the dead cells in hosts at 12 hpi. Apoptosis was visualized in *B. oleracea* at 6 hpi with DAPI (Beyotime, China). Leaf discs under inoculums were fixed with methanol-acetone (3:1) for 24 h at room temperature, and then treated with HCl (1 mol/L) at 60 °C for 8 min. After 5-min incubation with DAPI at room temperature, leaf discs were washed with PBS buffer (0.05 mol/L, pH 7.8) for three times. Tissues from the edge of leaf discs were viewed using a fluorescence microscope (Olympus, Japan).

### Antioxidant enzyme activity assay

Total antioxidant capacity was tested in the pathogen- and agar disc-inoculated leaf samples in rice, R and S *B. oleracea* at 0, 6 and 12 hpi by using a 2,2-azino-bis-3-ethylbenzothiazoline-6-sulfonic acid (ABTS) method, with three biological replicates. All the samples were immediately ground to fine powders in liquid nitrogen. Approximately 0.2 g powder of each sample was added to 10 ml cold phosphate buffer (0.05 mol/L, pH 7.8), and centrifuged (6000 r/min) for 15 min. The liquid was then tested using a Total Antioxidant Capacity Assay Kit (Beyotime, China) following the manufacturer’s instruction.

## Additional Information

**How to cite this article**: Mei, J. *et al.* Transcriptomic comparison between *Brassica oleracea* and rice (*Oryza sativa*) reveals diverse modulations on cell death in response to *Sclerotinia sclerotiorum. Sci. Rep.* **6**, 33706; doi: 10.1038/srep33706 (2016).

## Supplementary Material

Supplementary Information
