# Corrigendum: Transcriptomic comparison between *Brassica oleracea* and rice (*Oryza sativa*) reveals diverse modulations on cell death in response to *Sclerotinia sclerotiorum*

**DOI:** 10.1038/srep34900

**Published:** 2016-10-10

**Authors:** Jiaqin Mei, Yijuan Ding, Yuehua Li, Chaobo Tong, Hai Du, Yang Yu, Huafang Wan, Qing Xiong, Jingyin Yu, Shengyi Liu, Jiana Li, Wei Qian

Scientific Reports 6: Article number: 33706; 10.1038/srep33706 published online 09 20 2016; updated: 10 10 2016.

The original version of this Article contained a typographical error in the spelling of the author Huafang Wan, which was incorrectly given as Huafan Wan. This has now been corrected in the PDF and HTML versions of the Article.

