# Supplementary material for: Transcriptomic comparison between Brassica oleracea and rice (Oryza sativa) reveals diverse modulations on cell death in response to Sclerotinia sclerotiorum
Source: Sci Rep. 2016 Sep 20;6:33706. doi: 10.1038/srep33706 (PMC5028746; doi:10.1038/srep33706)

Transcriptomic comparison between *Brassica oleracea* and rice (*Oryza sativa*) reveals diverse modulations on cell death in response to *Sclerotinia sclerotiorum*

Jiaqin Mei[1], Yijuan Ding[1], Yuehua Li[1], Chaobo Tong[2], Hai Du[1], Yang Yu[3], Huafan Wan[1], Qing Xiong[4], Jingyin Yu[2], Shengyi Liu[2], Jiana Li[1], Wei Qian[1]*

Supplenmentary Table1 Summary of sequence read alignments to the reference genomes of *Brassica oleracea* and rice

Sample	Os0		Os6		Os12	
	Number	Percentage	Number	Percentage	Number	Percentage
Total Reads	31,325,474		35,918,740		35,210,272	
Total BasePairs	3,932,668,792		4,507,561,147		4,421,821,229	
**Map to Genome**						
Total mapped reads	25,021,122	79%	27,657,138	77%	25,924,464	73%
Unique match	24,581,025	78.00%	27,115,393	75%	25,426,827	72%

Continued

Sample	R0		R6		R12	
	Number	Percentage	Number	Percentage	Number	Percentage
Total Reads	26153966		26366740		24692942	
Total BasePairs	2353856940		2373006600		2222364780	
**Map to Genome**						
Total mapped reads	63	70%	18103863	69%	16989248	69%
Unique match	17258138	66%	16913098	64%	16079529	65%

Continued

Sample	S0		S6		S12	
	Number	Percentage	Number	Percentage	Number	Percentage
Total Reads	24904228		26296584		25114528	
Total BasePairs	2241380520		2366692560		2260307520	
**Map to Genome**						
Total mapped reads	18773809	75%	19990677	76%	18790793	75%
Unique match	17652882	71%	18915745	72%	17714157	71%

Supplenmentary Table 2 Common DEGs found in rice and *Brassica oleracea* during 0 to 12 hpi by *Sclerotinia sclerotiorum*

Arabidopsis homologous	RPKM								
	R0	R6	R12	S0	S6	S12	Os0	Os6	Os12
AT1G01060	321.0	30.1	154.8	229.5	31.3	15.6	1272.8	275.6	132.9
AT1G01070	2.7	11.1	9.3	5.0	8.8	17.4	2.9	33.6	9.5
AT1G01190	2.7	0.7	0.2	3.4	3.7	0.3	36.8	161.5	185.2
AT1G01580	13.7	11.0	10.2	18.5	9.2	5.7	5.3	4.6	0.8
AT1G01630	134.6	63.4	83.7	131.4	72.9	147.8	214.7	50.2	45.4
AT1G02335	56.2	38.1	38.3	50.4	21.8	21.9	2.2	7.1	4.2
AT1G02370	8.4	11.0	24.2	12.3	17.3	17.0	2.5	5.8	7.9
AT1G02470	2.4	7.8	4.7	7.5	21.3	47.4	107.2	31.0	27.1
AT1G02660	16.1	41.0	40.5	9.5	54.7	30.1	8.6	13.9	3.0
AT1G02860	4.2	6.4	10.2	9.1	9.6	24.0	31.6	8.5	7.1
AT1G03850	18.1	12.6	10.4	4.4	22.4	37.1	27.0	33.0	11.9
AT1G03870	96.5	20.5	8.0	93.1	103.7	42.6	5.2	10.1	3.6
AT1G03970	14.8	29.1	34.5	11.9	30.3	27.2	35.7	29.7	58.1
AT1G04000	48.4	49.3	40.2	33.1	65.0	55.9	27.3	14.3	9.5
AT1G04180	1.0	1.3	0.7	1.7	3.8	0.8	6.2	1.8	1.8
AT1G04280	12.4	33.6	28.7	18.3	29.4	37.9	86.4	68.0	52.0
AT1G04310	9.4	17.3	22.5	7.8	12.6	8.3	28.6	80.0	60.3
AT1G05200	15.9	12.7	11.6	15.8	13.3	6.8	38.7	17.8	13.3
AT1G05260	21.2	3.1	2.6	4.7	1.0	2.9	4.1	12.9	10.4
AT1G05850	341.6	162.8	165.5	355.4	282.2	139.5	12.7	45.8	53.0
AT1G05870	63.8	54.6	61.0	70.0	65.0	71.3	124.0	81.5	84.9
AT1G06040	93.3	44.0	61.7	102.0	45.6	14.1	3519.5	1811.6	1222.3
AT1G06180	17.3	2.5	4.8	7.2	1.9	14.5	0.4	2.8	4.7
AT1G06410	17.2	22.1	25.6	17.4	29.5	16.3	65.7	77.7	72.6
AT1G06570	76.2	738.9	1462.8	278.3	780.3	1772.0	73.4	15.2	7.4
AT1G06780	19.2	40.9	47.9	32.2	31.2	42.7	4.3	0.8	0.6
AT1G06950	258.8	228.0	296.2	222.2	214.9	173.9	298.3	152.7	55.2
AT1G07010	44.7	36.2	45.2	69.8	34.3	20.0	88.1	17.1	30.6
AT1G07180	16.7	17.4	11.0	42.3	27.3	26.1	33.6	8.8	9.2
AT1G07280	126.1	393.9	346.8	188.0	261.5	224.8	60.7	124.9	53.0
AT1G07420	17.6	20.0	12.4	13.2	22.3	9.2	140.9	38.8	34.4
AT1G07430	11.5	6.7	15.4	7.3	7.0	3.3	184.9	27.5	3.5
AT1G07520	2.4	3.1	3.3	3.0	3.0	3.3	132.9	46.3	44.9
AT1G07620	1.3	2.3	2.6	0.6	2.6	6.0	10.8	3.8	3.2
AT1G08380	1102.0	939.4	491.8	2951.5	2478.4	1935.1	2751.3	1748.1	400.0
AT1G08465	98.9	88.1	93.1	61.3	105.7	85.0	8.9	18.4	10.5
AT1G08570	79.0	149.1	132.4	144.8	137.0	102.3	636.3	208.7	137.8
AT1G08630	13.7	9.6	117.8	28.9	34.9	46.1	40.6	7.4	9.7
AT1G09210	127.9	109.8	99.1	80.5	53.0	42.3	61.1	193.4	255.3
AT1G09350	14.6	5.3	53.0	3.6	3.8	0.1	11.3	43.9	77.5
AT1G09460	11.5	23.0	21.6	7.6	21.4	19.8	0.5	4.2	2.6
AT1G09660	12.6	5.1	4.9	15.1	8.7	6.5	91.1	52.3	37.5
AT1G10020	38.0	31.9	36.7	30.0	41.7	29.9	11.1	18.1	9.7

AT1G10060	34.8	7.0	8.2	38.9	10.3	12.4	20.3	4.8	3.4
AT1G10360	20.2	8.7	14.7	30.3	6.3	4.4	517.0	181.0	126.9
AT1G10522	27.5	10.1	11.5	31.1	18.9	12.0	5.0	8.0	18.6
AT1G10660	10.7	22.3	12.5	12.9	20.2	14.0	41.9	11.0	5.0
AT1G10820	19.4	14.2	14.5	15.8	12.0	14.8	83.4	47.3	51.9
AT1G11530	8.8	46.5	51.2	23.3	75.2	104.6	126.1	45.5	52.1
AT1G11700	49.7	30.2	31.3	64.0	34.9	55.6	161.4	29.6	19.4
AT1G11960	12.4	5.7	4.3	3.2	4.9	4.3	54.4	46.0	52.0
AT1G12000	9.1	3.1	4.0	7.3	5.2	4.9	20.1	57.6	58.9
AT1G12040	2.6	2.2	1.9	1.0	0.9	0.3	3.7	4.5	1.5
AT1G12110	38.7	49.9	29.2	26.7	35.6	39.0	82.5	79.4	60.5
AT1G12240	55.2	34.2	51.2	100.9	34.4	33.4	7.3	21.0	13.1
AT1G12380	25.2	7.4	7.0	11.4	4.9	2.1	0.3	1.3	1.5
AT1G12710	1.7	14.7	7.3	2.0	21.9	13.4	219.7	190.7	175.4
AT1G12880	3.8	5.0	8.9	4.9	6.4	16.5	5.6	6.9	11.8
AT1G12900	508.5	302.1	218.7	813.8	517.4	217.6	626.1	320.5	88.8
AT1G13250	12.1	7.6	5.6	5.1	5.0	2.3	0.6	3.6	1.5
AT1G13260	144.9	228.0	225.5	153.0	273.0	171.6	174.3	147.7	140.2
AT1G13740	49.6	43.4	39.6	67.6	48.4	54.1	53.6	42.5	47.0
AT1G13750	5.1	5.3	3.3	4.2	3.7	1.8	18.8	69.1	71.2
AT1G13930	101.0	108.3	122.8	171.1	166.8	49.1	112.4	339.0	220.3
AT1G14390	0.3	0.7	1.0	0.1	0.8	1.0	1.7	6.3	2.8
AT1G14520	0.5	2.4	1.1	0.4	3.5	4.4	1.9	4.0	1.4
AT1G14700	38.8	12.1	9.8	51.6	12.9	14.9	56.5	12.7	11.4
AT1G14860	5.5	8.4	11.1	15.5	16.4	18.9	154.3	85.0	49.9
AT1G14870	269.2	418.7	344.1	142.6	290.3	699.6	77.7	288.4	358.3
AT1G15000	65.9	20.2	16.9	35.7	14.9	14.7	13.2	9.3	9.6
AT1G15380	4.7	7.5	180.7	3.2	16.1	50.2	10.0	99.6	22.9
AT1G15500	78.6	82.4	58.7	88.2	99.2	46.1	67.9	256.7	252.0
AT1G15520	3.5	8.6	7.2	1.0	1.3	5.6	4.1	13.0	11.0
AT1G15670	4.9	11.6	10.8	3.8	11.6	14.2	16.0	62.5	29.4
AT1G15690	209.2	412.3	912.5	349.0	506.2	445.6	226.0	466.7	328.5
AT1G15820	1760.6	1124.7	445.9	2020.2	1617.6	556.8	295.2	924.6	130.6
AT1G15980	86.6	85.1	41.2	85.9	86.8	70.7	17.6	27.4	70.6
AT1G16350	114.0	48.4	70.1	106.6	50.3	47.6	5.9	26.5	23.4
AT1G17050	16.4	8.7	8.9	22.1	10.6	10.0	45.1	16.4	15.2
AT1G17380	106.2	44.1	59.3	49.4	32.5	84.6	206.4	211.8	265.5
AT1G17430	2.5	6.4	6.0	2.0	7.3	4.0	10.1	3.3	1.1
AT1G17870	0.1	0.7	0.7	0.3	0.4	0.6	119.4	14.8	3.4
AT1G18250	40.8	4.5	2.8	14.8	2.5	1.9	0.1	2.0	0.3
AT1G18330	43.7	26.4	27.4	61.2	26.6	29.5	86.1	61.2	70.4
AT1G18480	7.2	11.3	9.7	5.9	8.8	9.2	0.6	4.9	4.4
AT1G18620	16.5	16.0	9.6	19.9	25.6	18.1	1.5	2.4	1.7
AT1G18670	4.5	4.8	1.7	2.9	3.3	1.6	14.3	36.8	36.6
AT1G18880	2.2	1.9	4.7	2.2	2.9	2.9	263.2	344.5	307.8
AT1G19150	51.4	16.1	3.6	76.6	41.8	9.6	13.5	25.4	55.4
AT1G19660	101.9	300.9	233.5	146.6	247.8	167.4	63.7	427.1	479.9
AT1G19670	122.1	19.2	7.5	55.8	13.2	7.9	45.3	15.9	18.6

AT1G19835	97.8	53.5	64.6	61.4	49.8	46.0	0.9	5.6	1.7
AT1G20020	358.7	451.2	271.6	435.9	434.3	171.1	1914.4	725.5	746.0
AT1G20640	21.3	32.1	56.3	25.6	38.4	46.5	31.2	10.6	7.6
AT1G20900	0.8	16.7	16.1	2.4	30.4	13.3	1.1	4.8	2.6
AT1G21210	2.6	0.9	2.7	0.3	0.9	0.5	0.6	5.4	9.9
AT1G21440	6.3	1.8	1.3	7.1	1.9	2.4	127.8	49.5	2.0
AT1G21460	100.8	110.2	95.7	145.1	90.3	111.7	240.4	288.7	126.3
AT1G21500	383.3	307.2	150.2	480.9	332.4	222.8	32.9	125.2	86.6
AT1G21560	8.0	2.7	3.0	6.4	6.1	5.3	48.1	24.7	29.3
AT1G21880	48.9	24.5	18.8	42.8	35.1	23.0	16.2	11.9	13.0
AT1G22070	40.1	75.7	66.1	31.4	77.9	50.7	113.0	37.2	30.3
AT1G22160	29.8	20.4	22.7	46.3	23.7	23.7	1332.1	511.1	473.3
AT1G22190	48.5	32.8	27.1	28.2	64.5	40.9	634.1	270.4	220.5
AT1G22640	13.3	34.2	55.0	19.5	26.3	57.1	14.1	46.9	40.8
AT1G22770	10.1	74.0	33.8	14.0	78.5	21.1	72.7	120.3	50.7
AT1G22850	79.0	57.5	63.8	83.6	52.8	33.6	77.0	23.2	21.7
AT1G23390	36.4	82.1	93.1	41.1	74.2	58.0	19.0	58.1	49.5
AT1G23710	34.0	46.9	62.6	31.3	98.0	135.8	138.5	72.8	59.0
AT1G23740	44.4	28.7	15.0	64.6	42.9	11.0	229.6	77.4	72.9
AT1G24140	6.4	14.4	7.7	2.3	10.8	12.7	5.2	6.2	5.6
AT1G24470	34.6	18.1	17.4	24.3	16.7	15.8	4.1	8.1	3.6
AT1G24530	16.3	39.0	39.4	10.0	48.4	53.2	0.4	3.1	2.4
AT1G25470	3.9	6.7	8.5	8.2	8.7	20.2	16.3	5.7	4.0
AT1G25510	2.7	0.7	0.8	2.0	1.9	1.2	2.0	5.4	1.5
AT1G26790	35.7	1.8	6.0	48.3	2.7	3.9	207.2	68.5	38.5
AT1G27100	22.8	16.7	17.5	22.5	52.5	53.5	5.0	11.0	16.1
AT1G27930	74.4	78.8	76.1	55.1	52.7	42.8	2.1	0.3	0.1
AT1G28050	33.8	113.6	92.0	32.6	117.4	75.9	7.7	65.3	47.3
AT1G28330	960.2	5083.2	4064.5	1482.2	5536.5	7341.1	862.0	2574.3	2956.0
AT1G28440	56.3	54.3	44.8	50.5	59.9	30.8	35.3	116.9	130.4
AT1G28570	46.1	34.1	29.5	59.9	38.1	33.8	20.8	15.6	12.9
AT1G29050	21.0	13.4	10.7	14.2	18.3	14.8	2.4	7.0	0.5
AT1G29900	16.8	14.5	16.0	16.7	15.8	6.5	24.2	73.1	54.7
AT1G29980	22.5	8.3	4.4	14.1	10.5	5.3	0.6	3.1	0.6
AT1G30230	245.6	131.2	128.2	152.8	155.4	172.7	187.7	302.2	443.7
AT1G30380	714.3	419.3	232.9	1111.1	645.3	436.0	4045.6	1441.2	415.4
AT1G30820	11.3	41.0	56.7	29.8	63.7	83.2	35.2	44.0	35.9
AT1G31350	15.3	8.1	14.8	18.3	10.6	34.9	11.1	1.4	0.4
AT1G32120	19.9	24.7	22.2	13.2	20.9	23.3	19.7	9.6	8.4
AT1G32350	18.5	79.5	95.7	18.0	50.0	126.4	56.5	15.7	12.1
AT1G32450	7.9	7.6	16.0	11.6	4.6	8.2	2.3	6.3	4.2
AT1G34340	16.4	7.8	7.1	9.5	4.4	1.8	22.9	10.9	9.6
AT1G44120	0.9	2.0	2.5	1.3	1.8	1.4	0.6	2.2	1.8
AT1G44446	94.6	41.1	67.4	124.5	56.2	25.9	372.4	165.0	62.0
AT1G44575	364.0	331.8	146.8	430.4	227.1	68.8	631.9	114.8	36.7
AT1G45201	93.0	86.9	47.4	126.2	54.0	55.2	166.2	34.0	13.1
AT1G45474	25.2	10.7	5.6	38.7	23.3	10.9	53.0	17.9	11.8
AT1G45688	183.4	84.7	79.6	110.9	81.8	75.8	15.3	34.8	30.5

AT1G47580	8.9	8.9	4.4	14.9	11.0	24.6	0.4	1.7	4.5
AT1G48260	1.8	1.8	1.1	2.0	3.3	0.7	502.4	224.9	248.5
AT1G49320	4.1	5.7	9.2	8.3	1.9	9.3	371.7	57.7	18.2
AT1G50420	33.1	45.6	45.0	25.0	35.0	17.7	119.1	110.4	34.8
AT1G50630	1.5	3.4	3.4	5.9	1.8	2.7	134.9	58.4	59.6
AT1G51140	45.3	20.2	46.8	82.3	20.5	40.8	248.5	364.0	397.2
AT1G51680	95.6	19.5	26.2	33.0	10.8	5.2	70.7	162.7	147.7
AT1G52720	6.0	12.7	13.7	20.0	40.9	65.7	174.5	317.0	849.9
AT1G53270	8.0	6.3	7.3	6.8	5.3	2.4	0.0	1.6	0.2
AT1G53300	9.2	6.0	4.0	7.7	7.1	2.6	1.0	6.2	1.3
AT1G53730	35.7	16.2	17.1	24.9	20.0	16.9	13.1	26.4	22.3
AT1G54050	11.0	6.5	1.8	31.2	10.0	6.7	18.7	2.0	1.6
AT1G55260	54.3	12.2	19.2	59.3	25.1	37.9	151.5	114.6	186.5
AT1G55450	39.6	73.4	50.9	27.1	83.1	72.2	21.6	8.3	5.2
AT1G55740	16.0	113.5	249.0	12.6	85.9	70.4	1.4	5.6	2.2
AT1G55850	20.6	39.9	39.8	18.3	30.6	37.4	75.8	38.8	30.6
AT1G56300	4.4	27.8	16.9	4.0	47.1	27.0	15.0	57.6	23.8
AT1G58180	24.4	86.0	125.9	70.0	136.6	232.0	187.7	50.2	32.6
AT1G59730	7.0	1.1	5.9	4.0	5.5	7.3	179.9	66.1	66.0
AT1G62250	6.8	4.3	3.6	5.0	5.6	2.4	694.9	90.0	27.0
AT1G62760	2.2	167.8	245.3	34.7	176.6	473.6	5.4	48.5	19.9
AT1G62770	86.2	34.0	24.2	59.3	31.3	28.8	11.5	17.8	15.0
AT1G63220	12.4	3.7	1.8	7.7	6.5	2.2	19.5	64.7	107.4
AT1G63840	30.8	33.9	23.9	23.8	42.5	45.4	86.0	35.4	22.5
AT1G64090	34.0	20.2	29.3	39.3	33.1	39.5	434.7	263.9	263.6
AT1G64500	74.0	19.3	36.3	99.8	59.8	50.4	494.1	148.5	105.0
AT1G64680	69.7	20.4	46.9	109.3	18.9	13.1	268.5	52.5	15.2
AT1G64780	6.1	3.5	14.8	13.3	6.6	3.8	348.2	131.2	146.6
AT1G64980	117.9	45.7	33.7	56.5	38.6	29.8	3.8	4.8	0.6
AT1G65060	9.5	0.2	0.3	5.2	1.1	0.6	2.3	5.0	0.1
AT1G67300	21.9	9.9	17.6	35.7	13.9	15.1	116.5	21.6	15.2
AT1G67370	3.2	8.1	6.4	3.8	6.3	4.1	17.0	6.5	5.6
AT1G67840	17.4	20.3	16.5	16.4	15.4	7.6	30.4	9.9	10.8
AT1G68560	69.5	31.4	21.4	39.9	40.4	13.6	0.1	1.1	0.1
AT1G68620	17.4	42.2	51.5	31.6	38.6	48.3	3.1	6.5	2.5
AT1G68810	15.1	20.0	24.3	14.3	14.7	20.0	6.0	11.5	8.5
AT1G69490	36.3	353.0	429.8	94.5	302.5	396.0	189.6	29.1	2.3
AT1G69520	4.0	0.7	2.1	1.1	0.9	0.8	36.3	13.1	12.0
AT1G69700	27.1	2.9	2.3	17.4	5.0	3.8	45.7	33.8	37.9
AT1G70300	6.9	11.0	14.3	11.0	13.4	11.7	17.9	63.6	74.2
AT1G70820	5.6	12.2	3.8	5.9	26.0	1.4	54.0	146.4	138.7
AT1G71692	3.1	0.2	3.1	3.0	2.1	2.7	36.4	6.2	3.3
AT1G71695	233.0	70.3	91.4	94.3	44.8	31.4	49.0	281.3	265.4
AT1G71870	0.6	1.5	3.3	1.3	2.8	1.2	9.3	10.6	21.3
AT1G72110	48.5	15.0	15.9	18.0	4.5	9.9	80.5	118.9	100.7
AT1G72180	58.9	97.5	68.1	43.9	60.5	30.4	74.6	17.9	12.9
AT1G72370	307.3	186.5	206.9	271.5	205.4	128.8	18.5	53.1	86.9
AT1G73590	9.2	5.9	4.9	5.8	5.4	2.3	22.5	11.5	5.8

AT1G73805	26.3	8.4	14.8	3.1	2.3	6.6	3.3	2.4	5.3
AT1G73850	4.1	9.0	7.9	2.9	5.5	7.8	0.1	5.9	2.7
AT1G74260	20.5	19.9	24.0	18.6	20.8	10.1	7.6	22.4	18.9
AT1G74360	6.2	12.1	11.6	3.8	10.0	9.3	6.5	25.5	29.0
AT1G74470	765.2	466.3	325.8	720.4	554.7	212.4	432.5	352.2	95.5
AT1G74730	49.2	27.6	12.5	63.4	34.8	28.8	1051.9	234.6	174.3
AT1G74790	6.2	7.8	9.9	9.5	11.2	8.2	2.9	22.8	44.7
AT1G74880	680.1	449.6	211.5	429.0	180.3	203.8	123.4	101.9	206.3
AT1G75130	4.3	1.8	1.5	6.8	3.3	2.5	47.1	21.8	30.0
AT1G75350	155.8	107.4	93.1	203.9	199.5	211.4	855.2	273.5	366.0
AT1G75390	13.8	3.3	5.8	4.5	2.9	3.1	325.0	106.6	97.9
AT1G75540	54.6	77.2	114.4	84.3	130.0	156.2	18.2	35.9	54.9
AT1G75690	44.2	29.4	24.6	68.3	39.8	28.6	20.3	28.6	12.3
AT1G75980	8.8	7.9	7.9	12.1	14.2	12.5	5.1	19.3	10.6
AT1G76080	484.7	320.8	374.6	516.3	470.3	172.6	393.6	688.9	156.1
AT1G76450	14.2	13.8	4.8	22.2	11.4	6.0	96.9	179.5	63.0
AT1G76570	5.2	10.4	8.5	17.3	25.6	35.1	177.2	47.4	29.5
AT1G77120	19.8	22.5	25.8	13.9	22.3	11.0	43.3	130.7	93.0
AT1G78240	63.0	113.6	139.6	85.5	111.1	114.7	0.9	2.8	2.4
AT1G78290	5.2	3.6	4.9	9.4	3.1	5.1	72.8	62.6	52.9
AT1G78600	9.2	11.5	10.4	8.7	17.0	10.6	56.2	205.5	118.1
AT1G79110	76.4	45.1	65.3	60.1	23.9	45.9	123.9	62.2	64.2
AT1G79600	32.1	88.6	83.8	60.2	112.3	53.4	194.6	73.3	38.7
AT1G79620	7.0	2.1	2.0	1.9	0.3	0.1	27.3	44.7	48.5
AT1G80050	16.7	5.8	6.0	10.6	5.0	6.3	73.8	105.5	121.8
AT1G80760	107.2	6.7	16.1	61.4	6.6	5.3	80.4	65.9	36.2
AT1G80840	40.3	39.9	43.5	28.8	83.4	112.4	11.2	37.5	41.4
AT2G01830	13.8	1.5	0.8	13.6	2.9	1.5	14.9	12.6	7.9
AT2G01918	8.8	3.0	2.1	8.1	4.4	4.7	14.0	11.6	32.4
AT2G02590	6.7	7.7	5.5	3.9	8.9	10.1	30.2	9.5	10.8
AT2G02710	50.9	325.2	360.5	97.2	299.7	314.7	22.9	55.7	26.8
AT2G03350	19.5	13.2	4.8	12.6	13.5	8.5	1.0	2.7	0.4
AT2G05620	225.1	125.8	122.1	352.4	179.3	199.9	630.5	110.0	51.1
AT2G05790	44.4	7.4	5.3	25.4	12.6	4.5	1.1	3.9	2.1
AT2G06850	88.1	17.9	28.4	85.7	106.8	74.9	0.0	4.7	0.0
AT2G15300	3.1	3.8	3.5	3.5	5.3	1.1	0.2	1.0	0.4
AT2G15440	14.8	2.3	1.6	4.8	1.6	1.9	19.1	26.2	16.6
AT2G16250	4.5	2.4	3.1	3.1	2.5	1.3	12.1	38.3	51.9
AT2G16660	12.5	4.0	5.0	10.8	4.5	5.5	4.5	23.5	25.7
AT2G17700	26.0	34.7	39.2	28.9	24.2	35.2	5.0	39.5	32.2
AT2G17730	8.6	3.0	2.8	3.5	3.3	1.3	92.7	92.4	88.8
AT2G17880	57.3	431.3	1090.4	169.3	642.8	1061.4	263.5	267.7	114.5
AT2G18540	0.6	0.9	2.2	0.4	0.3	0.3	3.1	0.4	0.0
AT2G19590	0.8	0.1	0.1	0.4	0.1	1.2	1.2	8.1	9.0
AT2G20670	13.1	50.2	106.3	22.5	67.0	113.7	24.5	7.7	2.9
AT2G20680	11.8	5.3	4.9	6.8	2.8	2.8	0.3	4.4	1.0
AT2G20900	6.4	10.6	6.3	0.8	2.3	5.0	77.6	74.0	69.6
AT2G21300	5.8	1.6	1.3	3.1	1.1	0.3	16.3	15.8	13.3

AT2G21320	23.7	2.8	11.6	30.4	6.2	2.0	268.7	53.0	27.8
AT2G21330	486.8	109.0	93.4	440.4	112.6	45.3	6736.4	1993.6	660.4
AT2G21410	26.7	18.8	24.7	41.2	21.7	13.4	43.9	45.6	37.2
AT2G21660	319.8	3346.5	2259.5	212.2	2190.0	1615.8	2328.8	8556.3	6665.9
AT2G21790	24.8	31.6	31.2	15.2	27.9	17.6	0.6	3.0	6.0
AT2G21960	14.9	15.8	3.8	13.9	10.5	7.1	93.2	37.6	47.5
AT2G22125	45.4	27.5	28.5	29.8	26.4	13.4	2.1	6.3	4.5
AT2G22240	7.4	3.5	5.9	7.6	3.4	1.6	165.2	47.6	29.3
AT2G22420	20.4	99.9	62.3	26.5	78.1	132.3	36.1	235.0	373.8
AT2G22540	206.6	107.3	119.6	238.0	135.3	177.6	150.8	181.6	191.2
AT2G22810	0.2	1.0	0.3	0.1	3.0	0.3	0.8	4.6	4.2
AT2G23290	4.3	1.4	0.7	0.9	0.6	2.3	49.6	48.6	35.0
AT2G23380	3.0	1.4	0.5	3.1	1.6	0.7	2.9	8.3	5.6
AT2G23790	5.2	7.5	5.9	7.6	8.1	3.5	110.0	125.5	125.9
AT2G23810	23.4	34.4	51.4	32.4	70.8	80.6	173.5	239.0	295.1
AT2G23840	12.7	39.5	30.3	23.7	44.1	21.6	95.2	97.5	32.1
AT2G24120	9.0	8.1	11.1	7.6	8.3	2.9	2.0	12.8	9.9
AT2G24150	6.5	18.1	23.3	5.5	16.8	14.1	5.1	6.2	0.8
AT2G25737	5.9	3.4	2.7	4.2	2.1	1.5	49.5	61.1	21.4
AT2G25770	2.6	3.2	2.6	1.2	1.7	5.2	193.1	59.3	22.8
AT2G25810	24.3	2.3	2.2	21.8	4.8	1.0	231.7	858.1	308.6
AT2G26695	34.4	17.5	7.2	23.4	45.7	68.5	24.3	8.8	10.4
AT2G26710	3.3	5.4	9.5	4.8	9.0	4.3	16.9	3.9	1.4
AT2G27500	38.5	50.2	56.0	32.3	48.5	75.6	8.3	43.3	38.9
AT2G27810	14.0	7.1	5.7	11.5	12.0	6.5	0.2	1.9	1.4
AT2G27830	41.0	116.5	137.6	78.7	68.1	257.0	81.5	19.2	18.8
AT2G28120	4.7	5.8	4.3	3.2	8.1	5.0	64.4	98.8	84.6
AT2G28250	18.7	8.2	8.2	13.4	9.4	7.5	6.8	7.9	3.6
AT2G28470	34.5	28.4	32.7	35.0	31.9	23.8	48.7	13.0	0.6
AT2G29130	28.6	5.3	4.4	13.2	3.3	2.4	12.5	2.0	0.0
AT2G29630	123.7	260.2	141.2	170.2	237.0	114.4	4.3	154.4	112.4
AT2G29650	23.2	14.6	27.2	32.9	26.9	10.3	109.6	146.1	67.7
AT2G29980	142.9	27.1	23.9	110.9	35.2	26.3	3.3	4.5	3.9
AT2G30490	121.5	44.3	106.7	78.5	32.3	39.5	96.0	49.0	31.4
AT2G31160	9.9	10.2	8.4	3.5	7.2	8.8	0.5	16.8	3.9
AT2G31810	40.2	62.4	69.9	47.6	74.1	83.8	6.0	50.9	38.9
AT2G32150	48.2	123.9	257.4	67.0	154.3	273.5	10.2	31.5	4.2
AT2G32290	4.9	5.6	4.4	10.7	7.3	3.3	85.5	14.9	12.2
AT2G32500	3.2	0.9	0.5	2.9	1.3	1.4	24.3	45.6	6.7
AT2G32530	18.3	8.1	9.0	27.5	11.0	6.2	68.3	73.0	71.0
AT2G32850	24.4	34.5	34.2	20.3	26.9	24.3	51.9	26.9	22.0
AT2G33210	25.0	17.8	19.7	22.7	20.3	12.6	11.7	36.4	52.4
AT2G33570	36.4	6.1	4.5	17.2	7.3	3.1	5.9	4.5	1.6
AT2G33735	16.5	7.9	9.3	20.8	14.8	21.4	8.0	6.6	1.8
AT2G34680	6.0	4.3	3.2	4.0	4.6	1.8	0.9	4.2	3.4
AT2G35120	16.5	9.3	20.1	12.4	6.3	11.2	13.3	25.2	28.4
AT2G35130	10.5	5.4	2.9	10.0	8.1	4.0	7.1	47.0	32.2
AT2G35710	8.7	19.5	16.2	1.5	18.4	18.1	12.7	45.7	37.4

AT2G35760	108.1	103.3	135.4	101.1	99.9	187.3	83.3	17.7	35.2
AT2G36010	8.2	6.9	4.1	6.0	6.4	4.2	37.9	17.7	18.7
AT2G36026	26.8	10.3	10.0	14.1	12.8	12.3	1.2	2.2	1.9
AT2G36630	31.3	20.9	43.8	59.1	30.8	23.7	104.1	34.0	15.2
AT2G36690	1.1	2.7	4.4	0.5	4.9	5.7	36.4	97.7	87.2
AT2G36830	227.0	74.2	139.6	259.4	162.4	238.5	404.5	131.0	40.2
AT2G36870	14.6	1.4	4.0	6.0	3.3	2.4	1.0	56.1	1.1
AT2G36890	7.3	5.8	10.9	2.2	4.9	9.2	10.0	21.5	25.6
AT2G36910	48.6	40.1	28.7	29.9	30.9	10.1	2.8	7.7	5.3
AT2G37040	78.8	15.3	18.8	31.4	6.5	6.5	177.1	412.4	317.0
AT2G37220	431.9	328.8	232.3	412.2	378.1	224.0	25.8	272.3	247.9
AT2G37540	8.7	7.5	6.1	17.0	9.0	8.8	9.1	2.0	0.5
AT2G37970	25.2	44.5	96.0	40.1	52.7	98.5	114.7	26.3	13.9
AT2G37980	10.5	10.9	8.4	11.9	9.5	16.0	43.3	11.7	8.1
AT2G38090	11.3	7.3	9.0	15.3	10.3	17.4	70.0	49.4	52.1
AT2G38170	110.7	160.5	352.1	248.0	240.5	263.2	608.8	113.6	97.5
AT2G38290	47.9	84.4	94.5	27.5	69.7	68.1	13.2	50.9	45.9
AT2G38310	67.9	57.3	43.1	45.5	78.2	129.0	29.2	296.7	310.2
AT2G38640	37.1	37.2	86.6	29.0	47.4	44.2	458.3	70.9	23.6
AT2G38750	20.2	0.5	0.9	11.8	0.5	1.0	4.3	25.3	27.2
AT2G38820	4.8	10.7	12.2	13.0	10.7	17.0	350.4	187.5	132.0
AT2G39560	3.7	0.7	0.6	2.4	2.5	1.2	1.3	4.4	0.9
AT2G39705	6.0	44.8	29.4	24.2	38.4	65.6	43.9	11.9	27.2
AT2G39730	5314.7	4331.9	4144.2	7364.8	5302.4	2695.9	28277.3	4681.4	797.3
AT2G39980	19.1	54.1	77.1	30.4	31.8	20.6	5.6	9.6	8.2
AT2G40540	62.5	54.8	37.5	52.4	51.8	21.0	93.3	56.1	34.5
AT2G40610	124.1	90.4	63.2	241.4	173.0	114.3	0.7	176.3	15.6
AT2G40900	11.7	23.5	23.7	15.8	17.8	24.1	113.3	62.2	59.7
AT2G41050	4.5	3.8	6.7	13.4	11.2	4.2	57.4	25.1	3.4
AT2G41120	6.8	2.4	4.3	10.9	4.7	1.3	33.1	28.0	6.8
AT2G41250	334.4	112.3	274.5	249.0	70.5	44.0	28.3	11.2	9.7
AT2G41290	3.1	5.5	4.8	5.2	6.1	9.2	0.9	1.9	0.2
AT2G41940	373.4	78.5	72.6	326.2	109.1	116.4	18.2	46.3	36.0
AT2G41950	12.6	5.6	4.5	20.3	11.3	12.4	21.7	8.0	9.1
AT2G42750	72.7	150.6	155.6	92.7	109.1	52.5	243.9	98.8	33.9
AT2G42910	69.3	73.5	72.0	44.7	50.5	24.4	9.7	32.0	53.9
AT2G43010	45.3	93.2	87.0	95.0	126.6	103.1	201.3	182.5	157.0
AT2G44670	167.8	168.8	134.0	320.3	241.3	772.7	148.5	294.0	408.2
AT2G45160	4.8	1.9	2.0	7.5	3.0	4.9	75.0	55.2	45.5
AT2G45470	239.4	51.7	41.8	153.1	65.8	43.4	0.5	2.7	1.0
AT2G46270	39.4	18.8	24.1	33.7	24.2	26.2	38.7	22.2	16.1
AT2G46600	231.3	205.9	473.5	416.5	243.8	554.8	212.5	70.5	106.9
AT2G46680	25.4	47.2	96.8	19.4	33.9	47.7	193.5	75.4	42.3
AT2G46790	6.3	11.7	46.3	9.7	10.0	0.7	62.7	176.9	39.6
AT2G46820	441.7	388.7	217.2	557.6	404.1	236.2	92.9	187.1	149.9
AT2G46940	23.2	10.3	13.1	30.7	13.9	21.2	7.2	2.0	0.7
AT2G47240	218.7	70.1	77.9	198.7	61.4	71.6	16.3	57.3	73.1
AT2G47460	9.6	4.3	4.3	4.6	6.0	4.1	5.4	5.7	0.5

AT3G01060	54.7	38.7	56.6	53.7	22.4	9.2	132.0	35.6	14.1
AT3G01420	71.6	32.4	23.6	86.2	38.8	58.2	108.2	161.4	75.2
AT3G01440	71.7	83.8	35.3	118.4	106.0	94.8	0.3	2.3	4.0
AT3G01470	318.6	553.1	573.9	434.8	423.4	793.9	966.9	773.6	798.6
AT3G01660	32.2	17.0	8.4	31.2	16.3	16.0	18.9	6.6	8.3
AT3G01990	37.9	84.9	47.3	40.3	62.7	35.6	36.6	96.0	69.2
AT3G02040	7.2	10.1	6.7	9.3	10.8	31.9	17.6	29.8	82.6
AT3G02380	260.3	60.5	155.5	494.2	68.1	131.6	154.4	29.9	6.1
AT3G03050	6.3	11.0	14.3	4.5	9.6	3.2	5.4	17.7	19.0
AT3G03150	117.8	173.2	215.3	364.7	344.3	623.5	322.0	479.6	981.9
AT3G03710	21.7	19.4	23.2	20.6	26.2	13.1	6.7	10.3	21.3
AT3G04600	43.4	25.5	32.5	35.5	38.7	33.6	15.0	37.6	40.9
AT3G05510	3.3	4.6	5.8	3.9	9.6	10.8	10.2	27.9	24.3
AT3G05690	30.0	22.4	21.6	33.5	20.8	12.4	14.5	7.3	8.3
AT3G05890	105.3	31.5	32.6	50.4	26.9	18.1	35.1	21.4	8.5
AT3G06390	6.1	1.6	1.2	0.2	1.1	0.7	3.7	12.2	18.8
AT3G06730	28.2	13.7	12.0	33.0	16.7	19.2	7.0	11.4	26.8
AT3G06868	84.3	64.7	56.1	66.9	51.1	25.8	1.6	1.4	0.5
AT3G07050	60.6	38.7	63.6	43.6	40.7	34.3	6.5	17.4	21.8
AT3G07340	3.2	0.8	1.6	2.8	1.8	1.6	41.6	78.3	35.8
AT3G07390	378.2	250.8	117.6	127.7	201.0	105.5	66.7	63.4	93.1
AT3G07650	65.5	165.3	165.6	118.9	158.2	256.3	17.9	32.7	87.4
AT3G07700	15.8	38.7	55.8	33.0	43.1	47.0	86.7	20.2	5.8
AT3G08570	8.0	3.7	3.0	5.8	4.6	3.4	12.5	2.1	0.2
AT3G09085	6.8	16.0	22.1	34.9	36.8	101.2	0.1	2.4	0.7
AT3G09430	2.5	2.6	2.8	3.9	3.2	0.7	12.5	2.7	1.0
AT3G10040	8.9	14.8	12.6	6.3	7.2	5.1	5.4	24.7	25.3
AT3G10050	125.6	46.3	43.9	87.5	65.1	33.3	6.2	37.7	39.5
AT3G10960	14.7	36.9	42.5	12.4	20.7	17.7	99.3	36.2	22.2
AT3G10985	88.0	172.8	143.7	195.8	215.1	371.1	173.8	167.9	74.3
AT3G11110	3.2	50.3	16.8	6.2	104.2	35.9	23.2	72.6	57.0
AT3G11600	8.8	2.6	16.6	8.4	3.6	5.7	87.9	53.7	26.5
AT3G11750	62.0	27.7	25.2	46.2	28.3	40.6	34.3	85.4	31.6
AT3G12090	13.9	23.5	15.4	17.5	17.8	30.4	15.6	27.7	16.0
AT3G12120	583.7	562.2	527.8	454.2	573.9	381.6	49.5	131.5	42.4
AT3G12145	157.0	5.6	12.3	28.7	2.1	2.7	0.1	10.6	2.5
AT3G12610	82.4	26.6	15.9	94.8	70.7	40.9	4.3	9.1	1.4
AT3G12710	68.1	29.0	36.6	56.8	43.2	41.8	0.0	2.7	0.1
AT3G13080	27.5	18.9	30.3	6.0	11.3	9.2	7.0	29.5	33.2
AT3G13120	325.9	238.3	157.0	331.6	243.4	198.0	113.8	44.0	77.5
AT3G13750	73.8	485.3	1010.0	173.7	460.0	477.3	2.6	18.7	2.7
AT3G14570	2.3	1.7	1.6	1.3	0.7	0.1	0.1	1.0	0.4
AT3G14770	10.7	8.1	9.5	27.7	10.0	23.4	89.5	32.5	35.6
AT3G14920	37.7	24.8	28.0	20.4	20.8	13.4	4.4	8.1	2.3
AT3G15353	2555.2	5435.4	4989.5	3176.8	7032.5	13852.9	34416.3	10373.5	11018.9
AT3G15450	103.3	2162.4	2401.3	246.0	1670.6	2240.9	43.7	391.8	247.2
AT3G15840	80.4	269.7	151.1	177.7	414.6	263.2	212.0	1168.2	714.5
AT3G16240	207.0	24.4	54.9	261.8	370.7	219.9	33.6	68.9	15.4

AT3G16250	140.6	88.7	48.1	130.1	110.9	117.4	7.3	187.6	297.1
AT3G17020	168.4	137.7	127.4	253.0	226.1	265.0	107.0	106.8	140.4
AT3G18390	79.1	66.3	69.6	79.4	70.8	42.1	5.6	34.7	23.5
AT3G19270	16.2	4.1	17.1	20.3	3.9	1.1	13.4	2.2	0.6
AT3G19450	59.2	27.3	30.3	45.8	29.4	23.9	16.7	9.3	22.9
AT3G19720	42.9	34.7	40.4	72.5	35.6	38.8	0.6	3.5	7.5
AT3G20570	145.1	100.1	120.6	125.5	66.0	83.0	8.0	3.3	0.6
AT3G20810	3.0	6.0	9.4	5.5	8.6	17.9	6.1	21.9	23.4
AT3G21180	12.5	17.5	17.7	15.7	13.8	5.4	60.7	72.7	67.3
AT3G21250	9.3	4.7	3.5	7.5	3.6	1.5	52.3	17.4	17.7
AT3G21330	2.0	18.8	2.5	2.4	28.6	2.0	4.6	20.7	8.2
AT3G21420	5.7	2.6	2.9	2.6	1.0	2.5	9.4	8.1	3.4
AT3G21560	51.0	30.1	45.9	57.3	25.7	21.1	554.4	74.0	45.2
AT3G21690	54.3	70.6	87.3	69.2	77.0	75.1	288.6	103.5	98.0
AT3G22530	34.7	49.8	64.2	84.0	103.5	149.9	16.1	13.3	6.1
AT3G22740	7.3	0.4	1.6	12.4	0.2	0.4	0.0	2.1	1.2
AT3G22840	65.6	18.2	77.3	48.0	8.1	4.1	1613.9	188.6	76.8
AT3G22880	13.8	53.8	35.2	8.9	74.4	83.6	17.0	5.7	3.9
AT3G23430	3.0	3.2	2.6	3.6	1.3	1.0	0.1	1.6	1.3
AT3G23580	4.3	1.5	4.1	3.8	3.9	5.6	0.1	1.8	3.5
AT3G23730	31.0	11.7	10.4	18.0	32.6	25.5	6.0	86.4	25.4
AT3G23740	2.4	1.5	1.3	1.5	0.4	0.3	3.3	0.7	0.2
AT3G23820	381.5	200.4	201.2	279.7	224.6	143.0	6.1	15.3	8.9
AT3G23920	83.1	64.4	119.6	94.0	73.7	52.1	165.9	32.9	17.7
AT3G23940	47.6	22.5	22.0	36.4	27.1	10.4	6.2	20.3	28.0
AT3G24190	52.0	32.7	44.7	52.3	23.4	12.7	355.4	84.9	51.5
AT3G24420	40.4	127.9	118.8	88.2	42.9	102.1	8.6	4.0	5.3
AT3G24460	3.7	6.6	10.0	9.2	13.0	13.2	185.0	26.7	9.9
AT3G25070	178.0	120.0	123.9	141.0	128.2	157.3	139.0	275.8	359.6
AT3G25660	32.5	23.2	18.6	19.0	18.4	5.1	16.7	17.0	36.3
AT3G26540	5.5	5.3	6.0	1.3	2.7	1.8	0.0	2.4	2.4
AT3G26740	4427.9	21464.9	17357.3	5224.9	13938.5	17458.0	320.9	3600.5	2433.4
AT3G26932	22.7	11.7	19.5	19.5	9.0	15.6	2.7	13.1	30.7
AT3G27250	35.5	24.6	27.0	53.0	18.2	12.9	11.0	2.3	0.1
AT3G27830	1652.3	942.8	643.2	810.6	696.3	594.6	7.8	4.9	14.8
AT3G28510	172.7	60.4	98.1	26.9	36.0	54.9	14.4	39.9	24.8
AT3G28960	0.9	1.7	5.0	2.8	1.8	2.2	60.2	25.8	22.0
AT3G29240	534.1	810.5	777.5	535.7	749.6	1106.7	804.7	140.2	96.6
AT3G29320	45.4	51.8	30.3	27.8	44.3	16.0	1.6	9.7	9.8
AT3G30180	36.4	34.8	19.5	73.0	43.9	32.2	7.0	4.2	1.8
AT3G30390	46.2	53.2	55.9	84.2	93.0	90.8	200.3	214.6	154.2
AT3G30775	22.4	152.4	219.8	24.6	138.9	211.0	129.3	37.4	30.0
AT3G43790	21.0	42.1	43.0	27.4	53.4	54.8	139.7	81.6	83.1
AT3G44350	25.6	14.6	15.7	8.4	15.0	12.6	3.7	13.7	22.6
AT3G45050	9.3	8.6	3.3	19.2	15.6	13.1	39.7	31.4	66.0
AT3G45090	4.1	2.0	1.6	2.8	0.9	1.1	0.4	1.9	3.2
AT3G45140	1445.5	297.4	505.9	700.1	78.1	53.5	279.8	125.4	31.1
AT3G46130	5.7	5.1	15.8	17.5	9.8	11.0	72.9	116.6	158.0

AT3G46640	17.1	60.4	66.0	34.3	70.2	104.4	20.7	87.3	70.7
AT3G47160	34.9	131.3	89.3	43.9	121.6	91.7	87.7	109.7	76.2
AT3G47470	2427.4	2383.4	1255.5	3962.0	3288.5	830.9	1487.7	2309.8	245.4
AT3G47570	1.3	4.6	1.2	2.1	3.0	2.2	3.6	7.9	7.0
AT3G48360	1.1	1.4	3.7	1.6	5.0	9.3	107.6	140.0	56.1
AT3G48530	106.2	183.1	261.3	228.0	252.5	396.4	24.2	96.4	75.9
AT3G48850	1.5	1.0	2.3	0.1	0.7	11.2	9.3	2.6	3.4
AT3G50040	2.1	4.9	4.5	2.3	5.5	5.3	49.2	32.0	33.1
AT3G51470	3.3	0.6	1.0	3.0	1.2	0.5	8.6	34.4	31.7
AT3G51550	127.0	95.2	103.9	96.5	94.1	62.8	13.6	33.9	31.7
AT3G51895	11.4	4.1	1.7	10.3	4.0	7.7	247.2	84.6	73.4
AT3G52150	159.2	67.6	48.0	163.4	107.6	67.6	10.0	16.6	49.7
AT3G52190	32.1	26.6	22.5	37.2	24.1	19.8	22.1	61.3	83.4
AT3G52490	11.4	8.8	7.6	7.9	5.8	4.6	9.2	5.1	4.5
AT3G52870	133.6	75.0	63.1	97.5	37.3	34.4	37.2	10.7	8.8
AT3G53830	4.7	2.7	10.7	2.7	4.1	0.9	34.8	6.1	2.0
AT3G53980	646.0	286.2	170.2	511.9	475.2	312.4	91.3	100.1	49.4
AT3G54000	31.7	37.1	35.2	20.8	37.2	29.8	139.5	54.8	53.1
AT3G54050	445.3	377.5	257.6	535.8	408.5	229.7	945.6	304.6	356.0
AT3G54500	205.8	95.3	175.2	212.6	116.7	39.3	81.6	13.4	3.0
AT3G54890	2637.4	2622.0	918.8	3220.9	3260.9	1701.0	1448.3	3513.7	636.9
AT3G55010	4.5	2.1	2.7	4.5	3.7	3.4	7.2	23.3	40.0
AT3G55120	24.1	13.0	13.8	25.5	12.2	12.9	27.0	41.8	15.0
AT3G55560	11.0	22.4	26.6	12.0	30.3	47.0	11.0	36.2	53.9
AT3G56290	29.3	6.2	12.3	55.1	11.3	6.7	405.8	44.4	3.1
AT3G56370	20.9	3.7	4.4	9.7	4.9	2.7	1.7	0.9	0.6
AT3G57150	78.1	54.5	76.7	49.0	53.6	36.7	14.2	36.0	35.2
AT3G57240	92.2	67.5	73.0	31.6	58.2	68.3	59.9	378.5	92.3
AT3G57520	54.9	161.4	263.7	119.4	186.2	511.8	853.7	281.7	318.8
AT3G57610	48.0	31.0	28.0	50.0	41.0	29.0	33.7	58.1	82.1
AT3G58060	1.6	0.6	0.5	1.9	0.4	0.5	105.1	19.9	13.5
AT3G59400	835.4	355.6	217.0	632.5	244.1	116.2	83.6	44.2	12.8
AT3G59420	7.0	3.1	4.4	8.1	5.5	5.1	0.2	0.5	0.1
AT3G59780	61.3	55.4	36.1	65.4	66.6	27.2	181.5	86.9	31.1
AT3G61470	2095.5	1989.3	1222.5	3205.3	2988.3	1513.7	1600.3	1327.5	261.6
AT3G61770	7.6	4.5	4.4	7.4	8.0	4.4	45.5	14.0	30.7
AT3G62110	3.8	3.0	1.1	5.4	7.2	4.5	8.8	14.7	24.5
AT3G62420	82.8	136.9	151.3	84.1	155.0	177.6	430.6	198.0	134.9
AT3G62550	318.1	866.7	460.8	457.1	778.2	1664.6	2141.6	6621.6	5278.7
AT3G62720	11.6	7.5	6.6	8.8	20.4	9.2	2.2	16.2	10.6
AT3G63120	59.2	26.0	27.7	36.9	25.6	23.3	2.8	11.1	5.9
AT3G63520	137.8	171.1	174.6	171.6	157.1	127.5	339.3	115.9	82.3
AT4G02280	3.8	9.1	17.8	16.3	14.9	7.4	35.4	218.2	141.4
AT4G02780	1.1	3.7	3.1	1.8	3.6	4.2	13.3	12.1	4.3
AT4G04020	56.4	77.0	151.6	213.7	168.5	181.5	738.2	251.3	168.4
AT4G05190	2.5	0.4	0.4	1.3	0.4	0.2	6.7	28.2	36.1
AT4G07960	5.5	3.9	3.3	4.9	2.4	1.3	5.1	11.6	5.8
AT4G09010	51.1	74.6	17.2	40.4	51.9	13.8	7.2	34.7	22.5

AT4G09350	27.7	5.8	12.1	85.1	15.1	17.7	849.1	213.4	154.7
AT4G09620	7.7	2.4	3.0	10.3	5.7	5.4	97.3	25.3	21.5
AT4G10310	4.7	5.5	7.2	5.2	7.7	6.7	18.5	5.1	2.6
AT4G10340	4194.5	2457.5	1472.0	5538.8	2938.0	1424.3	1084.1	384.1	14.0
AT4G10770	3.1	11.5	9.6	5.2	10.6	6.9	48.2	66.9	63.7
AT4G11910	0.5	0.3	1.5	1.4	5.7	6.2	162.3	25.1	5.4
AT4G12300	37.5	16.3	16.4	84.4	18.3	29.8	86.6	52.1	15.9
AT4G12730	203.0	134.5	139.7	103.1	144.5	84.1	7.5	13.2	6.3
AT4G13010	12.4	5.7	7.0	17.4	12.0	7.7	33.7	8.0	6.2
AT4G13040	27.1	13.5	11.0	19.7	11.4	14.9	202.1	43.1	26.3
AT4G14750	7.8	1.5	0.8	3.5	1.4	0.1	6.2	1.1	0.3
AT4G15440	31.4	9.7	5.1	14.9	11.1	0.9	89.9	15.8	12.4
AT4G15510	126.5	64.0	33.0	101.8	59.3	40.8	7.0	8.7	18.6
AT4G15530	30.8	123.6	434.6	55.0	128.2	201.2	403.6	126.6	77.1
AT4G16146	81.2	149.2	111.5	60.3	141.9	103.1	66.5	23.4	18.7
AT4G16563	22.0	309.0	293.4	50.4	202.6	145.5	3.6	0.5	0.1
AT4G17180	6.6	4.5	2.4	3.4	3.5	1.2	2.9	1.2	0.6
AT4G17260	45.3	75.5	61.4	16.1	28.3	23.0	6.6	21.6	23.4
AT4G17560	71.1	23.2	11.0	98.6	42.0	36.3	89.9	33.3	53.1
AT4G18290	1.5	4.8	3.2	5.8	8.4	8.5	18.5	8.4	8.2
AT4G18640	14.4	4.8	5.8	8.4	3.6	2.2	16.4	70.1	54.9
AT4G18750	2.4	1.7	1.7	1.3	1.4	0.3	2.1	3.7	7.5
AT4G18780	3.5	2.4	2.4	2.1	0.6	0.8	2.2	3.5	1.0
AT4G18810	65.6	50.6	64.6	102.7	54.1	12.9	105.7	36.9	20.2
AT4G19170	383.8	1435.3	1299.5	451.2	895.4	233.2	780.0	123.3	18.8
AT4G19230	0.7	5.6	7.4	1.1	9.6	3.6	16.3	50.1	47.4
AT4G21320	1.7	5.4	3.9	3.3	4.4	4.7	8.0	2.0	1.3
AT4G21445	26.7	26.6	11.5	36.6	34.1	33.2	12.4	61.8	86.4
AT4G21870	7.9	10.9	10.4	22.9	25.2	42.8	28.8	149.7	83.9
AT4G22010	4.8	2.2	0.5	10.1	2.6	0.5	0.2	1.6	0.2
AT4G22580	17.5	5.9	5.5	9.8	6.5	4.0	10.3	2.6	3.1
AT4G24120	61.9	88.2	82.7	57.5	68.0	68.7	30.6	63.7	41.0
AT4G24220	96.2	166.2	207.3	117.7	188.0	179.4	89.9	25.3	18.0
AT4G24380	16.4	3.6	5.0	17.7	5.1	9.6	486.6	95.7	46.8
AT4G24590	27.5	33.7	39.3	38.4	39.4	84.9	103.0	81.3	84.0
AT4G24660	11.0	9.5	3.1	12.3	11.2	7.8	11.6	9.6	11.1
AT4G25290	26.8	6.8	15.4	35.9	9.8	3.9	29.3	10.0	3.3
AT4G25500	391.5	384.0	516.2	327.8	305.0	331.1	59.3	241.0	111.5
AT4G25630	65.0	40.3	59.9	83.6	70.2	76.3	31.2	92.3	166.0
AT4G25700	36.4	14.4	15.1	73.7	29.7	22.7	119.3	38.3	26.1
AT4G25940	13.1	42.2	44.2	9.0	25.8	31.4	6.1	2.2	1.2
AT4G26130	73.4	114.0	102.8	51.7	118.6	39.3	87.5	13.1	3.7
AT4G26760	21.9	10.3	7.5	9.6	6.7	5.0	7.1	7.3	13.3
AT4G27280	68.9	40.6	29.5	30.2	76.9	114.0	53.6	19.1	12.0
AT4G27310	15.5	31.4	29.1	25.2	46.5	14.6	90.4	15.8	5.0
AT4G27350	0.3	2.4	4.6	2.6	4.0	2.4	47.4	8.8	3.3
AT4G27440	1076.2	433.9	198.8	661.7	412.5	160.9	0.6	48.7	11.8
AT4G27860	0.8	0.3	0.5	6.0	0.9	1.2	27.1	19.3	8.3

AT4G28040	50.8	37.8	120.2	65.3	69.6	139.8	40.3	16.7	4.4
AT4G28660	50.4	23.7	9.0	42.4	31.9	17.3	72.2	26.6	13.6
AT4G29310	5.3	1.0	0.4	5.3	1.2	1.0	1.2	2.1	0.0
AT4G29780	33.3	8.6	7.0	17.8	27.6	15.9	139.2	45.1	24.6
AT4G30810	122.0	49.2	48.5	139.0	65.3	38.0	11.2	46.9	35.3
AT4G30960	489.6	270.1	392.4	308.6	201.9	247.2	48.7	24.6	27.2
AT4G31120	33.9	35.8	33.4	48.2	60.4	73.0	3.3	8.2	9.1
AT4G31380	2.4	215.3	24.5	19.2	118.4	29.4	12.4	20.6	38.8
AT4G32190	36.4	36.0	59.8	46.4	41.1	24.0	28.0	10.6	12.6
AT4G32890	5.9	2.9	3.5	4.8	6.9	4.7	2.0	5.2	1.9
AT4G33040	60.5	38.5	18.4	120.8	59.9	75.0	276.3	66.6	34.6
AT4G33160	6.2	1.6	2.4	4.3	2.2	3.5	19.0	3.1	3.5
AT4G33420	27.6	31.9	40.6	25.4	52.7	54.4	10.1	19.9	40.4
AT4G34880	8.8	2.1	1.7	4.8	3.0	1.6	2.9	4.2	8.5
AT4G35770	1.6	198.3	353.7	112.9	468.6	1563.1	0.2	6.0	1.4
AT4G35790	63.1	147.0	118.5	65.5	146.7	81.0	9.8	10.9	6.2
AT4G36250	156.7	74.3	55.8	172.5	106.8	105.2	6.5	8.3	2.2
AT4G38540	1.4	6.8	10.5	1.5	9.8	27.9	16.6	18.6	10.6
AT4G38890	7.9	4.7	5.6	6.4	6.5	3.0	5.4	14.2	16.6
AT4G39710	167.5	106.9	36.3	140.4	101.5	95.5	18.4	22.1	51.8
AT4G39840	64.2	29.2	26.9	37.6	29.5	17.1	0.2	1.4	0.7
AT5G01220	39.0	84.4	67.6	34.6	62.5	33.1	97.9	43.6	51.8
AT5G01830	3.0	7.9	9.6	2.9	4.6	6.1	18.3	35.0	29.0
AT5G02810	41.8	115.7	83.6	40.2	118.1	39.5	93.1	36.0	8.0
AT5G02890	43.6	11.2	10.2	35.7	7.0	5.6	2.2	6.8	4.6
AT5G03170	12.1	2.0	3.4	5.7	1.5	1.2	9.1	3.9	0.8
AT5G03730	30.6	52.6	46.4	31.0	51.5	28.9	57.6	188.8	152.5
AT5G03760	29.9	2.1	2.7	18.4	2.8	1.9	0.2	0.4	1.4
AT5G04885	11.9	8.1	6.5	12.4	7.1	4.6	2.9	42.7	18.6
AT5G05170	238.4	164.9	153.4	217.1	194.5	132.8	2.9	8.8	4.5
AT5G05930	17.5	38.5	33.9	22.0	31.5	35.7	5.9	48.2	32.5
AT5G06570	30.4	66.8	63.4	10.7	30.1	26.1	148.0	118.3	124.8
AT5G07020	72.5	54.7	36.5	75.0	99.8	33.9	354.6	83.0	36.0
AT5G07070	9.3	14.4	15.6	9.0	19.6	15.5	181.6	145.0	110.0
AT5G08180	73.6	37.6	37.4	86.8	78.5	93.0	57.5	67.6	98.3
AT5G08350	0.2	0.7	2.2	0.7	2.9	3.7	34.4	27.6	19.0
AT5G08610	49.6	36.6	47.4	49.2	38.1	29.5	19.7	46.2	98.5
AT5G09530	12.3	0.8	2.0	0.7	0.4	0.2	6.8	61.8	22.2
AT5G09760	94.6	59.6	39.5	45.7	48.1	24.8	5.7	14.9	10.0
AT5G09930	4.0	2.4	5.3	2.9	1.9	1.1	1180.1	230.9	99.5
AT5G10930	74.7	53.2	36.9	32.3	34.6	13.2	62.6	17.7	25.6
AT5G11260	57.2	21.1	42.2	60.8	25.9	51.1	49.1	24.6	8.6
AT5G11890	117.5	81.0	85.7	72.6	82.4	63.0	24.3	15.6	9.1
AT5G11950	14.1	12.1	13.3	22.5	9.0	17.6	124.5	70.5	29.1
AT5G12140	314.6	379.6	303.6	280.5	256.3	292.4	228.0	587.0	552.3
AT5G12300	9.7	3.2	5.7	5.4	2.7	1.9	19.6	7.2	4.6
AT5G12950	49.9	34.8	30.0	36.9	42.2	23.9	20.9	19.6	23.6
AT5G13190	44.7	61.6	42.8	65.4	95.8	146.8	11.9	31.5	21.2

AT5G13500	34.7	31.1	29.7	29.3	23.8	24.4	9.6	14.2	14.1
AT5G13630	320.9	161.3	146.4	225.3	96.5	30.4	88.6	167.8	55.8
AT5G13930	33.6	5.5	5.0	47.6	6.2	4.1	8.6	58.1	7.2
AT5G14180	3.8	5.6	15.9	10.6	16.7	23.8	0.3	2.1	1.3
AT5G14360	17.4	6.2	3.2	11.9	5.7	12.1	0.0	11.0	2.5
AT5G14580	2.9	4.7	8.0	1.8	6.1	3.2	3.2	8.4	7.9
AT5G14700	4.4	1.6	2.6	3.5	3.1	4.1	22.2	18.3	8.9
AT5G14760	8.1	3.1	6.0	5.6	4.6	4.6	24.4	9.6	9.1
AT5G14910	105.4	26.8	61.9	86.4	53.6	60.9	23.3	26.9	63.8
AT5G15120	0.4	2.4	0.4	0.8	3.1	2.2	10.4	19.3	8.7
AT5G15250	0.5	0.5	0.1	2.2	0.8	0.4	4.3	0.9	0.1
AT5G15550	11.6	7.8	19.6	11.9	15.0	10.9	5.6	17.2	19.9
AT5G15780	699.7	127.5	186.9	397.3	235.5	229.7	3.1	1.8	0.1
AT5G15802	21.7	9.0	8.1	20.7	14.8	20.9	35.1	33.4	67.1
AT5G16010	80.9	50.3	49.1	93.0	49.9	33.2	164.7	32.2	12.5
AT5G17230	164.4	91.1	73.0	228.6	101.4	130.6	163.6	68.1	53.8
AT5G17420	6.0	2.8	4.0	2.0	0.8	0.4	1.4	5.6	0.6
AT5G18460	44.7	11.5	13.7	33.2	15.6	21.5	0.2	2.4	0.3
AT5G18670	53.8	49.6	69.9	81.0	52.8	60.2	120.0	37.7	35.4
AT5G18860	12.5	17.2	5.7	30.4	10.8	16.8	39.2	45.9	16.6
AT5G19530	48.1	111.0	80.0	50.6	96.0	72.6	0.3	2.6	4.4
AT5G19850	14.9	14.0	10.7	20.0	12.9	4.9	29.9	11.7	16.0
AT5G20140	17.5	40.5	32.8	19.8	15.6	8.6	145.0	30.7	6.4
AT5G20250	13.6	206.4	567.1	39.9	297.4	360.3	9.8	38.2	21.2
AT5G20720	256.3	116.7	110.9	430.8	236.7	229.6	89.2	118.5	245.0
AT5G20935	81.5	56.9	39.5	60.6	57.0	92.4	7.0	10.4	23.6
AT5G21100	57.8	5.7	8.3	41.5	3.6	2.5	0.7	3.9	0.8
AT5G21170	101.3	402.7	528.8	361.3	649.7	1185.0	71.0	129.0	113.6
AT5G22920	4.6	34.5	89.4	17.7	54.6	86.1	14.3	57.5	30.3
AT5G23050	24.2	80.4	126.5	99.2	147.0	205.7	43.6	176.5	114.8
AT5G23240	27.8	423.8	251.1	52.5	302.4	264.7	2.6	51.2	75.7
AT5G23340	13.5	10.2	15.2	16.0	15.2	19.7	2.5	2.4	0.1
AT5G23530	12.4	40.8	24.4	4.7	17.3	12.8	78.6	86.9	61.4
AT5G23810	1.4	1.0	2.0	0.1	1.0	0.4	11.7	16.6	20.4
AT5G24000	9.9	10.7	10.5	13.6	8.8	14.2	11.5	2.2	0.5
AT5G24120	58.3	42.1	61.9	65.7	36.4	23.8	518.0	95.5	27.8
AT5G24270	3.4	3.9	0.9	1.5	1.8	0.7	17.0	6.7	11.3
AT5G24300	27.4	22.7	24.7	24.8	24.4	10.5	23.5	6.2	6.7
AT5G24530	454.3	216.6	223.6	126.9	106.7	74.3	3.8	30.9	7.7
AT5G24770	501.2	0.4	1.8	20.9	0.9	0.2	92.0	69.9	21.4
AT5G24800	67.1	60.4	64.7	45.4	34.7	51.7	72.6	41.1	38.7
AT5G24850	4.2	1.2	1.5	4.9	2.7	3.5	10.2	3.2	1.6
AT5G25610	1454.2	938.0	711.0	573.8	284.4	129.2	4.3	2.7	0.0
AT5G25770	10.1	10.3	16.3	7.1	6.9	5.1	58.7	17.3	9.5
AT5G25930	32.5	85.1	56.3	18.7	79.0	79.8	133.5	198.9	230.5
AT5G26340	9.5	9.9	14.3	1.1	6.1	7.1	120.2	44.1	65.9
AT5G26920	49.3	23.2	37.7	13.8	22.0	32.2	11.2	92.1	55.0
AT5G28300	38.2	35.4	15.8	30.7	52.7	24.2	0.0	0.9	0.3

AT5G28750	79.8	49.3	43.4	145.4	83.3	108.6	33.4	228.2	185.6
AT5G35630	696.1	527.3	331.2	780.7	606.4	424.7	603.6	261.1	104.5
AT5G35970	29.8	25.0	26.5	43.3	27.4	7.2	96.7	25.6	12.9
AT5G39000	119.7	123.4	117.4	114.0	139.5	197.4	48.8	92.6	82.6
AT5G39660	112.1	18.7	42.0	117.8	18.3	35.3	72.0	40.3	21.3
AT5G40500	18.8	25.2	15.7	17.4	34.9	33.2	39.1	29.5	5.6
AT5G41040	5.0	2.0	1.1	2.3	2.6	3.3	1.0	7.3	3.3
AT5G42070	64.0	29.9	27.7	86.5	64.8	49.7	141.3	27.7	7.3
AT5G42370	0.1	0.5	0.3	0.0	0.8	0.6	2.8	1.4	3.8
AT5G42390	130.7	200.5	193.8	178.6	213.0	135.4	43.4	182.3	98.3
AT5G42760	3.1	0.7	0.8	8.0	2.4	1.7	41.3	11.6	16.1
AT5G43150	118.8	67.1	56.7	256.1	101.8	101.0	149.6	69.4	88.8
AT5G44030	7.4	3.8	4.5	4.3	1.2	1.1	2.9	4.7	1.0
AT5G46800	73.2	15.0	20.1	98.1	23.2	19.2	520.7	121.3	40.2
AT5G46910	5.2	2.6	1.6	2.7	3.3	4.4	4.5	7.4	4.4
AT5G47040	31.2	99.3	74.6	50.7	76.6	53.2	104.0	279.7	225.9
AT5G47240	2.9	12.4	29.0	7.5	29.1	41.9	11.4	18.2	2.5
AT5G47560	107.4	79.2	72.5	102.4	96.4	62.4	119.8	248.6	46.2
AT5G48310	0.7	0.1	0.1	0.4	0.1	0.1	2.4	0.4	0.3
AT5G48485	12.8	7.0	9.1	112.0	51.4	107.2	35.7	107.0	30.7
AT5G48790	24.0	17.5	21.4	49.7	35.5	17.5	1.9	16.1	6.4
AT5G49480	24.8	11.5	18.7	11.3	7.1	13.7	238.9	49.4	40.0
AT5G49940	64.3	99.3	105.8	137.8	193.7	208.6	237.6	78.8	68.8
AT5G50200	59.4	105.5	110.3	44.2	88.9	114.6	1.4	7.9	8.4
AT5G50570	8.5	8.0	4.0	9.5	10.5	8.9	9.8	9.4	5.2
AT5G53730	135.9	303.0	223.5	84.0	109.0	157.8	9.6	25.2	30.0
AT5G54080	48.0	193.9	405.1	126.8	263.2	581.0	78.1	30.0	24.0
AT5G54270	606.8	636.7	250.0	951.7	1124.1	151.0	1875.0	486.5	50.1
AT5G54770	957.9	2020.5	1822.7	1403.7	1640.9	1040.6	146.1	1189.3	256.2
AT5G55630	29.5	17.5	21.7	35.2	24.0	24.6	148.8	24.3	11.9
AT5G56840	71.1	34.2	27.8	34.7	20.7	32.9	365.9	161.0	182.8
AT5G56850	24.9	24.0	37.4	22.1	27.6	63.4	43.7	16.5	7.0
AT5G56890	38.7	23.2	22.7	20.3	14.8	8.6	70.9	25.7	10.2
AT5G57170	24.1	22.2	5.1	51.9	23.6	21.8	13.2	9.2	38.4
AT5G58070	183.9	379.5	296.4	179.9	319.7	284.0	233.2	90.2	98.5
AT5G58560	24.5	18.0	13.6	21.8	26.5	19.6	86.1	45.0	52.5
AT5G60600	170.4	192.4	202.6	245.4	216.8	99.0	515.3	159.0	137.2
AT5G60720	2.7	2.1	1.9	1.4	0.4	0.5	1.9	0.4	0.4
AT5G60920	183.2	93.0	85.8	112.7	79.2	42.3	6.6	14.8	12.5
AT5G61380	58.8	320.1	380.9	102.3	260.5	344.4	13.1	49.0	25.7
AT5G61510	74.1	74.8	75.6	50.6	32.7	61.0	2.6	0.4	1.0
AT5G62190	72.8	47.9	60.5	101.6	70.9	49.5	22.8	64.1	67.1
AT5G63100	27.3	9.8	10.6	11.3	5.1	8.0	9.1	3.2	1.8
AT5G63380	13.3	7.9	9.3	15.6	6.9	4.3	67.3	100.2	68.1
AT5G64170	23.4	40.4	35.6	59.4	79.1	34.8	244.4	54.6	31.5
AT5G64530	9.9	30.1	24.6	14.7	25.9	36.8	5.5	0.7	0.3
AT5G64810	87.7	44.8	57.6	16.1	34.8	38.9	43.6	116.7	110.5
AT5G66170	15.1	4.9	11.8	30.7	15.7	9.8	13.4	24.8	24.1

AT5G67030	206.4	393.5	470.7	312.0	369.2	183.1	74.7	52.9	13.6
AT5G67370	26.2	24.5	33.5	86.0	42.2	27.8	593.8	131.6	46.7
AT5G67390	5.7	9.3	16.6	12.5	13.2	8.5	1.5	34.8	14.7
AT5G67550	4.7	2.5	2.6	3.8	1.5	1.4	0.5	4.2	1.8

Supplenmentary Table 3 Significant GO and KEGG enrichments in rice and *Brassica oleracea* upon up- and down-regulated DEGs

Category	Term	Count	%	P-Value	List Total	Pop Hits	Pop Total	Fold Enrichment	Bonferroni	Benjamini	FDR
**By 2166 up-regulated DEGs in rice**											
GOTERM_BP_FAT	GO:0019748~secondary metabolic process	26	1.9	2.709E-09	567	96	8363	3.995	1.379E-06	1.379E-06	3.911E-06
GOTERM_BP_FAT	GO:0055114~oxidation reduction	118	8.7	2.785E-09	567	1025	8363	1.698	1.418E-06	7.088E-07	4.021E-06
GOTERM_BP_FAT	GO:0006979~response to oxidative stress	26	2.2	2.711E-07	567	152	8363	2.911	1.380E-04	4.599E-05	3.913E-04
GOTERM_BP_FAT	GO:0009765~photosynthesis, light harvesting	10	0.7	4.163E-07	567	17	8363	8.676	2.119E-04	5.297E-05	6.010E-04
GOTERM_BP_FAT	GO:0009698~phenylpropanoid metabolic process	16	1.2	7.745E-07	567	51	8363	4.627	3.941E-04	7.884E-05	1.118E-03
GOTERM_BP_FAT	GO:0006575~cellular amino acid derivative metabolic process	21	1.6	2.667E-05	567	107	8363	2.895	1.348E-02	2.260E-03	3.849E-02
GOTERM_CC_FAT	GO:0005576~extracellular region	48	3.5	1.226E-13	409	273	7887	3.391	1.275E-11	1.275E-11	1.370E-10
GOTERM_CC_FAT	GO:0048046~apoplast	19	1.4	9.081E-06	409	108	7887	3.392	9.440E-04	4.721E-04	1.015E-02
GOTERM_CC_FAT	GO:0005618~cell wall	19	1.4	1.961E-05	409	114	7887	3.214	2.037E-03	6.794E-04	2.192E-02
GOTERM_CC_FAT	GO:0030312~external encapsulating structure	19	1.4	3.559E-05	409	119	7887	3.079	3.695E-03	9.250E-04	3.979E-02
GOTERM_CC_FAT	GO:0019898~extrinsic to membrane	14	1.0	4.281E-05	409	69	7887	3.913	4.442E-03	8.900E-04	4.785E-02
GOTERM_MF_FAT	GO:0016684~oxidoreductase activity, acting on peroxide as acc	33	2.4	1.877E-08	693	164	10565	3.068	7.977E-06	7.977E-06	2.641E-05
GOTERM_MF_FAT	GO:0004601~peroxidase activity	33	2.4	1.877E-08	693	164	10565	3.068	7.977E-06	7.977E-06	2.641E-05
GOTERM_MF_FAT	GO:0016209~antioxidant activity	33	2.4	2.096E-07	693	181	10565	2.780	8.909E-05	4.455E-05	2.950E-04
GOTERM_MF_FAT	GO:0005506~iron ion binding	77	5.7	3.190E-06	693	687	10565	1.709	1.355E-03	4.518E-04	4.489E-03
GOTERM_MF_FAT	GO:0043169~cation binding	238	17.6	3.122E-05	693	2904	10565	1.249	1.318E-02	3.312E-03	4.393E-02
GOTERM_MF_FAT	GO:0043167~ion binding	238	17.6	3.269E-05	693	2905	10565	1.249	1.380E-02	2.775E-03	4.598E-02
KEGG_PATHWAY	osa00940~Phenylpropanoid biosynthesis	14	1.0	9.455E-07	148	37	1977	5.054	7.942E-05	7.942E-05	1.015E-03
**By 1304 down-regulated DEGs in rice**											
GOTERM_CC_FAT	GO:0016021~integral to membrane	54	7.22	1.892E-06	187	1196	7887	1.904	1.589E-04	1.589E-04	2.031E-03
GOTERM_CC_FAT	GO:0031224~intrinsic to membrane	55	7.35	1.897E-06	187	1229	7887	1.887	1.593E-04	7.967E-05	2.036E-03
**By 630 up-regulated DEGs specific to R *B. oleracea***											
GOTERM_BP_FAT	GO:0009628~response to abiotic stimulus	71	11.5	8.985E-11	367	1197	13998	2.262	8.724E-08	8.724E-08	1.414E-07
GOTERM_BP_FAT	GO:0006979~response to oxidative stress	17	3.06	6.408E-06	367	287	13998	2.614	6.470E-03	5.408E-04	1.014E-02
GOTERM_BP_FAT	GO:0009314~response to radiation	30	4.85	2.700E-05	367	482	13998	2.374	2.587E-02	8.700E-03	4.249E-02

GOTERM_BP_FAT	GO:0010033~response to organic substance	55	8.89	3.165E-05	367	1176	13998	1.784	3.026E-02	7.653E-03	4.980E-02
**by 875 down-regulated DEGs specific to R *B. oleracea***											
GOTERM_BP_FAT	GO:0009617~response to bacterium	23	2.94	1.796E-05	460	244	13998	2.868	1.775E-02	8.916E-03	2.837E-02
GOTERM_BP_FAT	GO:0010033~response to organic substance	66	8.44	1.952E-05	460	1176	13998	1.708	1.928E-02	6.468E-03	3.083E-02
GOTERM_CC_FAT	GO:0033279~ribosomal subunit	49	6.27	2.292E-20	460	294	13779	4.992	4.562E-18	4.562E-18	2.871E-17
GOTERM_CC_FAT	GO:0022626~cytosolic ribosome	50	6.39	9.859E-20	460	317	13779	4.725	1.962E-17	9.810E-18	1.235E-16
GOTERM_CC_FAT	GO:0005840~ribosome	61	7.8	1.045E-19	460	470	13779	3.888	2.080E-17	6.933E-18	1.309E-16
GOTERM_CC_FAT	GO:0044445~cytosolic part	43	5.5	4.214E-17	460	271	13779	4.753	8.386E-15	2.097E-15	5.278E-14
GOTERM_CC_FAT	GO:0030529~ribonucleoprotein complex	67	8.57	1.336E-15	460	671	13779	2.991	2.651E-13	5.307E-14	1.665E-12
GOTERM_CC_FAT	GO:0043232~intracellular non-membrane-bounded organelle	87	11.1	3.671E-13	460	1144	13779	2.278	7.306E-11	1.218E-11	4.598E-10
GOTERM_CC_FAT	GO:0043228~non-membrane-bounded organelle	87	11.1	3.671E-13	460	1144	13779	2.278	7.306E-11	1.218E-11	4.598E-10
GOTERM_CC_FAT	GO:0005829~cytosol	64	8.18	6.646E-13	460	708	13779	2.708	1.323E-10	1.890E-11	8.324E-10
GOTERM_CC_FAT	GO:0015934~large ribosomal subunit	29	3.71	3.295E-12	460	173	13779	5.021	6.557E-10	8.196E-11	4.127E-09
GOTERM_CC_FAT	GO:0005618~cell wall	53	6.78	4.189E-10	460	611	13779	2.598	8.337E-08	9.263E-09	5.247E-07
GOTERM_CC_FAT	GO:0030312~external encapsulating structure	53	6.78	7.081E-10	460	620	13779	2.561	1.409E-07	1.409E-08	8.868E-07
GOTERM_CC_FAT	GO:0022625~cytosolic large ribosomal subunit	23	2.94	9.686E-10	460	138	13779	4.992	1.927E-07	1.752E-08	1.213E-06
GOTERM_CC_FAT	GO:0022627~cytosolic small ribosomal subunit	20	2.56	6.386E-09	460	114	13779	5.255	1.271E-06	9.775E-08	7.998E-06
GOTERM_CC_FAT	GO:0015935~small ribosomal subunit	21	2.69	1.584E-08	460	133	13779	4.730	3.152E-06	2.252E-07	1.984E-05
GOTERM_CC_FAT	GO:0005730~nucleolus	32	4.09	2.279E-07	460	332	13779	2.887	4.535E-05	3.024E-06	2.854E-04
GOTERM_CC_FAT	GO:0031981~nuclear lumen	35	4.48	2.034E-05	460	472	13779	2.221	4.039E-03	2.529E-04	2.547E-02
GOTERM_MF_FAT	GO:0003735~structural constituent of ribosome	56	7.16	5.959E-20	492	394	14806	4.277	2.545E-17	2.545E-17	8.391E-17
GOTERM_MF_FAT	GO:0005198~structural molecule activity	60	7.67	3.876E-16	492	538	14806	3.356	1.422E-13	7.105E-14	4.663E-13
**by 940 up-regulated DEGs specific to S *B. oleracea***											
GOTERM_BP_FAT	GO:0010200~response to chitin	27	3.39	1.656E-14	440	127	13998	6.764	1.481E-11	1.481E-11	2.577E-11
GOTERM_BP_FAT	GO:0009743~response to carbohydrate stimulus	30	3.77	4.504E-12	440	199	13998	4.796	4.031E-09	2.016E-09	7.016E-09
GOTERM_BP_FAT	GO:0010033~response to organic substance	73	9.17	2.329E-08	440	1176	13998	1.975	2.084E-05	6.947E-06	3.627E-05
**by 828 down-regulated DEGs specific to S *B. oleracea***											
GOTERM_BP_FAT	GO:0016051~carbohydrate biosynthetic process	27	3.57	5.553E-07	477	254	13998	3.119	6.217E-04	6.217E-04	8.900E-04
GOTERM_BP_FAT	GO:0009628~response to abiotic stimulus	74	9.79	5.792E-07	477	1197	13998	1.814	6.485E-04	3.243E-04	9.284E-04
GOTERM_BP_FAT	GO:0009250~glucan biosynthetic process	14	1.85	1.274E-06	477	75	13998	5.478	1.426E-03	4.755E-04	2.042E-03

GOTERM_BP_FAT	GO:0009791~post-embryonic development	57	7.54	1.427E-06	477	851	13998	1.966	1.597E-03	3.994E-04	2.287E-03
GOTERM_BP_FAT	GO:0005976~polysaccharide metabolic process	24	3.17	1.621E-06	477	219	13998	3.216	1.814E-03	3.630E-04	2.598E-03
GOTERM_BP_FAT	GO:0034637~cellular carbohydrate biosynthetic process	22	2.91	1.861E-06	477	189	13998	3.416	2.082E-03	3.473E-04	2.982E-03
GOTERM_BP_FAT	GO:0000271~polysaccharide biosynthetic process	16	2.12	3.212E-06	477	107	13998	4.388	3.591E-03	5.138E-04	5.148E-03
GOTERM_BP_FAT	GO:0044271~nitrogen compound biosynthetic process	39	5.16	4.043E-06	477	506	13998	2.262	4.518E-03	5.659E-04	6.480E-03
GOTERM_BP_FAT	GO:0006073~cellular glucan metabolic process	17	2.25	4.595E-06	477	124	13998	4.023	5.133E-03	5.716E-04	7.364E-03
GOTERM_BP_FAT	GO:0033692~cellular polysaccharide biosynthetic process	15	1.98	9.832E-06	477	103	13998	4.274	1.095E-02	1.101E-03	1.576E-02
GOTERM_BP_FAT	GO:0044042~glucan metabolic process	18	2.38	1.490E-05	477	151	13998	3.498	1.655E-02	1.516E-03	2.388E-02
GOTERM_BP_FAT	GO:0044264~cellular polysaccharide metabolic process	18	2.38	1.932E-05	477	154	13998	3.430	2.140E-02	1.801E-03	3.095E-02
GOTERM_CC_FAT	GO:0044435~plastid part	79	10.4	1.658E-13	453	984	13779	2.442	3.879E-11	3.879E-11	2.131E-10
GOTERM_CC_FAT	GO:0044434~chloroplast part	77	10.2	2.775E-13	453	954	13779	2.455	6.495E-11	3.247E-11	3.568E-10
GOTERM_CC_FAT	GO:0009507~chloroplast	171	22.6	1.644E-12	453	3192	13779	1.629	3.847E-10	1.282E-10	2.113E-09
GOTERM_CC_FAT	GO:0009536~plastid	171	22.6	1.034E-11	453	3259	13779	1.596	2.420E-09	6.050E-10	1.330E-08
GOTERM_CC_FAT	GO:0009570~chloroplast stroma	44	5.82	3.072E-11	453	420	13779	3.187	7.189E-09	1.438E-09	3.949E-08
GOTERM_CC_FAT	GO:0009532~plastid stroma	45	5.95	4.530E-11	453	442	13779	3.097	1.060E-08	1.767E-09	5.823E-08
GOTERM_CC_FAT	GO:0009526~plastid envelope	45	5.95	1.791E-10	453	461	13779	2.969	4.191E-08	5.987E-09	2.302E-07
GOTERM_CC_FAT	GO:0009941~chloroplast envelope	42	5.56	1.592E-09	453	440	13779	2.903	3.726E-07	4.658E-08	2.047E-06
GOTERM_CC_FAT	GO:0031975~envelope	55	7.28	4.116E-08	453	751	13779	2.228	9.630E-06	1.070E-06	5.291E-05
GOTERM_CC_FAT	GO:0031967~organelle envelope	54	7.14	8.024E-08	453	745	13779	2.205	1.878E-05	1.878E-06	1.032E-04
GOTERM_MF_FAT	GO:0046527~glucosyltransferase activity	17	2.25	1.086E-05	487	137	14806	3.773	6.493E-03	6.493E-03	1.603E-02

Continued

KEGG enrichments

ID	Term	Nr. Genes	% Associated Genes	Term P-Value	Term P-Value Corrected with Bonferroni
**R-specific up**					
**KEGG:04146**	**Peroxisome**	**19**	**15.079364**	**1.993E-05**	**2.990E-04**
KEGG:00630	Glyoxylate and dicarboxylate metabolism	6	5.3097343	5.229E-03	7.843E-02
KEGG:00960	Tropane, piperidine and pyridine alkaloid biosynthesis	4	7.6923075	6.151E-03	9.226E-02
KEGG:00740	Riboflavin metabolism	2	20	8.353E-03	1.253E-01
KEGG:00350	Tyrosine metabolism	4	6.6666665	1.017E-02	1.525E-01
KEGG:00950	Isoquinoline alkaloid biosynthesis	3	8.823529	1.210E-02	1.815E-01
KEGG:00450	Selenocompound metabolism	3	8.1081085	1.524E-02	2.286E-01
KEGG:00905	Brassinosteroid biosynthesis	2	13.333333	1.861E-02	2.792E-01
KEGG:00590	Arachidonic acid metabolism	2	9.090909	3.840E-02	5.761E-01
KEGG:00130	Ubiquinone and other terpenoid-quinone biosynthesis	3	5.6603775	3.908E-02	5.863E-01
KEGG:00380	Tryptophan metabolism	3	5.6603775	3.908E-02	5.863E-01
KEGG:03020	RNA polymerase	3	5.5555553	4.097E-02	6.146E-01
KEGG:00250	Alanine, aspartate and glutamate metabolism	4	4.3010755	4.285E-02	6.428E-01
KEGG:00592	alpha-Linolenic acid metabolism	2	4	1.584E-01	1
KEGG:00906	Carotenoid biosynthesis	2	4.6511626	1.243E-01	1
**R-specific down**					
**KEGG:03010**	**Ribosome**	**56**	**14.043584**	**2.907E-22**	**6.687E-21**
KEGG:00260	Glycine, serine and threonine metabolism	7	7.7777777	3.037E-02	6.986E-01
KEGG:00053	Ascorbate and aldarate metabolism	5	8.77193	4.089E-02	9.406E-01
KEGG:00040	Pentose and glucuronate interconversions	4	4.9382715	3.486E-01	1
KEGG:00073	Cutin, suberine and wax biosynthesis	3	8.823529	1.035E-01	1
KEGG:00196	Photosynthesis	2	5.4054055	3.523E-01	1
KEGG:00230	Purine metabolism	10	4.484305	3.388E-01	1
KEGG:00250	Alanine, aspartate and glutamate metabolism	4	4.3010755	5.541E-01	1

KEGG:00360	Phenylalanine metabolism	6	4.6875	3.245E-01	1
KEGG:00400	Phenylalanine, tyrosine and tryptophan biosynthesis	4	4.7619047	3.652E-01	1
KEGG:00410	beta-Alanine metabolism	3	6.122449	2.245E-01	1
KEGG:00510	N-Glycan biosynthesis	3	4.477612	4.906E-01	1
KEGG:00562	Inositol phosphate metabolism	6	6.6666665	1.258E-01	1
KEGG:00640	Propanoate metabolism	2	4.1666665	6.749E-01	1
KEGG:00750	Vitamin B6 metabolism	2	14.285714	7.777E-02	1
KEGG:00906	Carotenoid biosynthesis	2	4.6511626	6.547E-01	1
KEGG:00940	Phenylpropanoid biosynthesis	7	4.9295774	3.370E-01	1
KEGG:03008	Ribosome biogenesis in eukaryotes	8	5.16129	2.503E-01	1
KEGG:03410	Base excision repair	3	5.769231	2.514E-01	1
KEGG:04070	Phosphatidylinositol signaling system	5	5.263158	2.501E-01	1
KEGG:04075	Plant hormone signal transduction	16	4	4.706E-01	1
KEGG:04145	Phagosome	7	4.516129	3.644E-01	1
**S-specific up**					
**KEGG:04626**	**Plant-pathogen interaction**	**18**	**7.4380164**	**1.367E-06**	**1.914E-05**
KEGG:00480	Glutathione metabolism	8	5.714285818	6.085E-03	6.519E-02
KEGG:04075	Plant hormone signal transduction	16	4	8.457E-03	1.184E-01
KEGG:04130	SNARE interactions in vesicular transport	5	7.352941	1.164E-02	1.630E-01
KEGG:03020	RNA polymerase	4	7.4074073	2.292E-02	3.208E-01
KEGG:04140	Regulation of autophagy	3	8.333333	3.521E-02	4.929E-01
KEGG:04145	Phagosome	7	4.516129	3.653E-02	5.115E-01
KEGG:00430	Taurine and hypotaurine metabolism	2	11.764706	4.499E-02	6.299E-01
KEGG:00230	Purine metabolism	9	4.0358744	4.541E-02	6.358E-01
KEGG:00290	Valine, leucine and isoleucine biosynthesis	2	5.1282053	1.851E-01	1
KEGG:00592	alpha-Linolenic acid metabolism	2	4	2.671E-01	1
KEGG:00906	Carotenoid biosynthesis	2	4.6511626	2.146E-01	1
KEGG:00920	Sulfur metabolism	3	5.882353	8.285E-02	1
KEGG:02010	ABC transporters	2	6.8965516	1.151E-01	1
**S-specific down**					

**KEGG:00196**	**Photosynthesis**	**7**	**18.918919**	**4.328E-05**	**1.125E-03**
KEGG:00900	Terpenoid backbone biosynthesis	6	10.169492	4.515E-03	1.174E-01
KEGG:00500	Starch and sucrose metabolism	11	5.4726367	2.177E-02	5.661E-01
KEGG:00400	Phenylalanine, tyrosine and tryptophan biosynthesis	6	7.142857	2.372E-02	6.168E-01
KEGG:00071	Fatty acid metabolism	3	4.477612	2.627E-01	1
KEGG:00100	Steroid biosynthesis	2	5.263158	2.676E-01	1
KEGG:00195	Photosynthesis	5	6.0240965	6.907E-02	1
KEGG:00230	Purine metabolism	9	4.0358744	1.982E-01	1
KEGG:00250	Alanine, aspartate and glutamate metabolism	5	5.376344	1.006E-01	1
KEGG:00260	Glycine, serine and threonine metabolism	5	5.5555553	9.050E-02	1
KEGG:00270	Cysteine and methionine metabolism	7	4.827586	1.098E-01	1
KEGG:00350	Tyrosine metabolism	3	5	2.130E-01	1
KEGG:00380	Tryptophan metabolism	3	5.6603775	1.657E-01	1
KEGG:00450	Selenocompound metabolism	2	5.4054055	2.577E-01	1
KEGG:00600	Sphingolipid metabolism	2	7.4074073	1.602E-01	1
KEGG:00630	Glyoxylate and dicarboxylate metabolism	6	5.3097343	1.260E-01	1
KEGG:00640	Propanoate metabolism	2	4.1666665	3.659E-01	1
KEGG:00710	Carbon fixation in photosynthetic organisms	8	5.3691278	6.154E-02	1
KEGG:00790	Folate biosynthesis	2	7.4074073	1.602E-01	1
KEGG:00906	Carotenoid biosynthesis	2	4.6511626	3.171E-01	1
KEGG:00909	Sesquiterpenoid and triterpenoid biosynthesis	2	8.333333	1.324E-01	1
KEGG:00910	Nitrogen metabolism	3	4.6153846	2.483E-01	1
KEGG:00920	Sulfur metabolism	4	7.8431373	4.566E-02	1
KEGG:00941	Flavonoid biosynthesis	3	8.571428	6.482E-02	1
KEGG:00966	Glucosinolate biosynthesis	2	5	2.875E-01	1
KEGG:02010	ABC transporters	3	10.344828	4.057E-02	1
KEGG:03030	DNA replication	4	5	1.627E-01	1

Supplenmentary Table 4 Transcriptome changes for genes involved in antioxidation, Ca^{2+} signalling and WRKY transcription factors

Brassica oleracea

Gene ID	*Arabidopsis homologue*	Description	RPKM value						Log2(Fold change)			
			R0	R6	R12	S0	S6	S12	R-6	R6-12	S-6	S6-12
**Antioxidation related**												
Bol026381	AT2G28190	CSD2 (COPPER/ZINC SUPEROXIDE DISMUTASE	35.0	14.1	28.3	96.8	53.0	34.2	-1.31	1.01		
Bol044394	AT3G56350	superoxide dismutase (Mn), putative	1.3	7.0	1.0	4.3	2.7	5.8	2.40	-2.75		
Bol029708	AT1G12520	ATCCS (COPPER CHAPERONE FOR SOD1)	74.9	795.5	355.5	351.2	230.4	304.6	3.41	-1.16		
Bol037061	AT4G18360	(S)-2-hydroxy-acid oxidase; peroxisomal, putative	5.1	8.6	22.1	4.2	7.1	6.3		1.35		
Bol006556	AT1G08570	ACHT4 (ATYPICAL CYS HIS RICH THIOREDOXI	21.6	45.8	38.6	34.2	57.3	52.2	1.08			
Bol044748	AT2G22420	peroxidase 17	18.1	96.8	58.6	44.9	74.7	66.2	2.42			
Bol015837	AT5G64120	peroxidase, putative	0.1	1.6	6.6	0.2	0.3	0.1	3.49	2.04		
Bol026246	AT1G73680	pathogen-responsive alpha-dioxygenase, peroxidase act	21.9	44.6	35.6	30.3	24.2	30.2	1.03			
Bol039416	AT1G73680	pathogen-responsive alpha-dioxygenase, peroxidase act	1.6	4.2	2.3	3.7	2.1	1.2	1.38			
Bol014449	AT3G63080	ATGPX5 (glutathione peroxidase 5)	8.6	27.0	16.2	17.4	19.2	32.4	1.65			
Bol040405	AT3G13750	BGAL1 (Beta galactosidase 1),peroxidase	38.2	324.4	731.8	224.7	362.5	381.3	3.09	1.17		
Bol005945	AT3G13750	BGAL1 (Beta galactosidase 1),peroxidase	35.6	160.9	278.2	48.9	97.5	96.0	2.18			
Bol025454	AT1G20630	CAT1	36.1	111.9	811.8	123.6	119.5	106.6	1.63	2.86		
Bol013716	AT4G35090	CAT2	3.7	4.7	14.9	7.8	7.9	13.1		1.68		
Bol034639	AT4G35090	CAT2	48.7	53.8	145.1	296.8	55.4	79.1		1.43	-2.42	
Bol024259	AT4G35090	CAT2	300.5	167.5	347.0	627.7	169.3	184.4		1.05	-1.89	
Bol026973	AT1G20620	CAT3	27.0	69.1	69.4	60.0	67.3	41.5	1.35			
Bol040250	AT5G36940	CAT3	4.0	17.3	17.6	20.2	27.7	21.2	2.11			
Bol003831	AT3G03720	CAT4	3.2	8.2	8.6	6.3	11.1	10.8	1.38			
Bol010903	AT2G02390	ATGSTZ1	1.9	12.2	20.8	5.3	7.5	6.0	2.66			
Bol006068	AT2G02930	ATGSTF3	56.3	137.2	164.0	12.5	81.5	65.6	1.29			2.40
Bol016517	AT2G02930	ATGSTF3	893.5	2332.2	1658.6	103.1	556.9	975.0	1.38		2.43	
Bol033291	AT2G29480	ATGSTU2	1.0	2.2	6.9	2.0	8.2	9.8		1.63	2.04	
Bol033290	AT2G29470	ATGSTU3	0.3	3.1	5.7	0.7	0.5	15.3	3.35			4.80
Bol023071	AT3G09270	ATGSTU8	0.7	0.5	0.4	0.1	1.9	1.9			4.29	
Bol009790	AT1G17170	ATGSTU24	28.4	28.5	75.9	6.0	3.1	15.3		1.41		2.28

Bol039366	AT1G74590	ATGSTU10	0.5	0.5	3.0	0.4	0.5	1.6		2.53		
**Ca^{2+} signaling related**												
Bol032583	AT3G25600	calcium ion binding	8.9	16.2	12.6	10.6	28.9	15.0			1.44	
Bol032916	-	calcium ion binding	4.4	4.1	4.2	9.0	5.0	14.4				1.51
Bol012150	AT5G39670	calcium-binding EF hand family protein	11.7	4.2	3.3	8.3	12.4	29.8	-1.48			1.27
Bol010002	-	calcium-binding EF hand family protein	47.9	38.5	58.9	96.3	65.0	130.7				1.86
Bol043000	AT3G29000	calcium-binding EF hand family protein	3.9	1.6	1.1	2.6	4.9	26.8				2.46
Bol006771	AT5G39670	calcium-binding EF hand family protein	3.8	0.7	1.1	1.4	1.1	3.8	-2.55			
Bol024879	AT3G47480	calcium-binding EF hand family protein	37.4	10.5	13.2	8.6	4.3	6.6	-1.83			
Bol036465	AT5G39670	calcium-binding EF hand family protein	25.7	8.7	7.3	8.9	6.6	11.6	-1.56			
Bol013114	-	calcium-binding EF hand family protein	-	0.8	1.6	0.6	0.7	1.4	9.69			
Bol023625	AT1G24620	calcium-binding pollen allergen, putative	1.5	5.6	3.9	0.5	3.3	4.7	1.94			
Bol028357	AT4G20780	calcium-binding protein, putative	7.9	8.8	3.6	3.5	11.2	29.4		-1.30	1.68	1.39
Bol024333	AT4G20780	calcium-binding protein, putative	78.8	314.0	102.1	29.8	102.5	174.5	2.00	-1.63	1.79	
Bol029597	AT2G46600	calcium-binding protein, putative	42.7	41.8	52.5	28.2	45.7	109.0				1.26
Bol023797	-	calcium-binding protein, putative	13.1	1.4	5.1	1.4	0.6	-	-3.23	1.88		
Bol030794	AT1G18210	calcium-binding protein, putative	102.9	47.0	30.0	65.3	105.1	85.5	-1.13			
Bol002750	AT3G19100	calcium-dependent protein kinase, putative	3.4	1.8	2.9	6.3	2.1	1.9			-1.62	
Bol030994	AT3G19100	calcium-dependent protein kinase, putative	10.4	5.5	6.6	14.2	5.2	7.4			-1.46	
Bol003626	AT3G19100	calcium-dependent protein kinase, putative	9.0	3.8	2.1	10.5	4.2	0.6	-1.25		-1.33	-2.86
Bol026232	AT1G73805	calmodulin binding	18.0	5.3	9.4	0.6	1.3	3.2	-1.77			1.27
Bol039988	AT1G73805	calmodulin binding	8.3	3.2	5.4	2.5	1.0	3.4	-1.38			1.87
Bol023302	AT3G22930	calmodulin, putative	65.3	49.0	38.2	14.6	29.6	50.5			1.23	
Bol027610	AT1G76650	CALMODULIN-LIKE 38 (CML38)	10.7	9.4	6.5	13.6	35.2	60.2			1.37	
Bol002305	AT3G50770	calmodulin-related protein, putative	30.2	41.6	47.6	10.4	19.2	70.7				1.88
Bol010737	AT3G01830	calmodulin-related protein, putative	5.4	3.4	4.3	0.8	2.8	17.3				2.63
Bol028942	AT4G37010	caltractin, putative	31.4	115.1	180.0	74.6	112.7	192.3	1.87			
Bol004202	AT2G27030	CAM5 (CALMODULIN 5)	96.5	116.8	121.8	134.3	315.3	334.7			1.23	
Bol020573	AT2G27030	CAM5 (CALMODULIN 5)	25.8	45.6	35.6	24.2	119.1	84.6			2.30	
Bol044303	AT2G27030	CAM5 (CALMODULIN 5)	68.7	32.1	34.2	37.8	40.5	37.4	-1.98			
Bol016716	AT5G26920	CBP60G (CAM-BINDING PROTEIN 60-LIKE.G)	30.8	9.4	19.0	4.5	10.7	11.7	-1.71	1.17	1.27	
Bol012758	AT5G26920	CBP60G (CAM-BINDING PROTEIN 60-LIKE.G)	3.8	2.0	4.9	0.4	1.7	1.6		1.35	2.22	

Bol035298	AT5G42380	CML37 (CALMODULIN LIKE 37)	11.6	3.9	2.1	3.3	2.8	15.3	-1.59			2.44
Bol007568	AT4G04720	CPK21	3.1	6.6	4.4	9.5	10.6	33.2				1.65
Bol032679	AT4G04710	CPK22	3.2	2.4	2.6	1.3	1.2	2.7				1.23
Bol027148	AT5G66210	CPK28	25.7	15.3	10.6	9.2	20.2	12.9			1.13	
Bol007592	AT3G57530	CPK32 (CALCIUM-DEPENDENT PROTEIN KINAS	17.4	45.0	53.6	17.1	33.2	21.2	1.37			
Bol005351	AT5G37770	TCH2 (TOUCH 2)	154.0	62.6	51.8	44.1	93.1	72.0	-1.30		1.77	
Bol030676	AT2G46450	ATCNGC12	13.5	9.3	41.2	0.5	6.4	2.3		1.61	3.82	2.36
**WRKY**												
Bol020064	AT2G03340	WRKY3	7.7	5.2	5.6	10.5	2.5	2.0			-2.48	
Bol007231	-	WRKY3	18.1	34.6	35.5	18.1	29.9	13.5				-1.14
Bol010361	AT1G13960	WRKY4	11.8	26.9	36.3	11.0	18.1	9.8	1.20			
Bol030382	AT1G62300	WRKY6	9.9	42.2	32.7	8.3	18.7	30.8	2.93		1.16	
Bol006350	AT5G46350	WRKY8	3.0	1.6	4.5	0.3	0.6	0.8		1.50		
Bol017448	AT4G31550	WRKY11	16.7	20.5	18.8	9.3	27.1	35.8			1.55	
Bol033735	AT4G31550	WRKY11	45.0	34.2	21.4	15.1	53.9	46.3			1.83	
Bol028817	-	WRKY13	1.5	0.6	2.2	1.8	0.5	2.2				2.19
Bol041910	AT2G24570	WRKY17	27.5	22.5	20.4	15.7	23.7	51.1				1.20
Bol033747	AT4G31800	WRKY18	23.7	7.6	15.2	5.4	12.4	18.4	-1.63		1.20	
Bol017463	AT4G31800	WRKY18	20.3	11.8	24.5	2.3	5.0	9.0		1.53		
Bol017960	AT4G31800	WRKY18	18.1	3.3	5.3	3.6	2.9	4.0	-2.46			
Bol011480	AT4G01250	WRKY22	17.3	22.3	16.3	6.7	29.9	27.7			2.16	
Bol002625	AT2G30250	WRKY25	2.3	4.6	6.3	1.5	5.6	7.4			1.85	
Bol033331	AT2G30250	WRKY25	17.5	32.8	51.0	15.0	32.0	33.5			1.96	
Bol024531	AT5G07100	WRKY26	64.1	75.6	75.4	15.4	37.4	21.2			1.28	
Bol037073	AT4G18170	WRKY28	2.3	6.8	6.2	3.9	14.0	15.4	1.57		1.83	
Bol042105	-	WRKY29	3.3	1.6	0.2	1.3	-	1.3		-3.33	-1.39	1.37
Bol022456	-	WRKY30	1.9	6.9	3.2	-	1.2	4.7	1.90	-1.12	1.23	1.96
Bol036212	AT5G24110	WRKY30	3.6	13.3	6.8	0.4	3.5	4.9	1.87		3.15	
Bol028485	AT4G22070	WRKY31	0.9	0.3	1.4	0.2	0.1	0.5		2.37		
Bol020355	AT2G38470	WRKY33	59.3	121.9	121.8	26.6	129.3	117.4	1.39		2.28	
Bol035694	AT5G22570	WRKY38	43.6	1.7	6.8	3.9	0.4	1.8	-4.66	1.98	-3.46	
Bol036034	AT5G22570	WRKY38	34.0	12.6	11.7	6.1	7.9	14.8	-1.43			

Bol038569	AT1G80840	WRKY40	15.0	14.2	18.3	15.9	35.8	33.2			1.18	
Bol040536	AT1G80840	WRKY40	24.5	25.1	24.7	11.4	47.0	76.9			2.46	
Bol021481	-	WRKY40	0.8	0.6	0.4	1.5	0.6	2.4				2.15
Bol011769	AT4G11070	WRKY41	7.7	14.2	10.7	4.8	15.3	39.8			1.67	1.38
Bol030560	AT4G04450	WRKY42	2.3	7.0	4.0	2.3	3.3	2.4	1.62			
Bol013898	AT3G01970	WRKY45	14.5	109.1	132.7	30.3	100.6	271.4	2.91		1.73	1.43
Bol000927	AT2G46400	WRKY46	60.2	26.3	36.0	19.4	25.1	40.1	-1.19			
Bol011419	AT4G01720	WRKY47	16.8	93.0	60.7	4.1	9.8	14.7	2.47		1.27	
Bol001371	AT5G49520	WRKY48	2.1	5.9	5.1	0.5	1.1	0.9	1.46			
Bol005635	AT5G43290	WRKY49	3.8	1.1	1.0	5.0	3.0	0.7	-1.79			-2.14
Bol012741	AT5G26170	WRKY50	38.1	14.0	14.0	4.5	7.9	11.4	-1.44			
Bol022304	AT5G26170	WRKY50	8.8	3.9	5.5	3.4	4.5	4.3	-1.18			
Bol015902	AT5G64810	WRKY51	38.5	15.4	26.6	7.4	17.4	22.1	-1.32		1.23	
Bol019066	AT5G64810	WRKY51	23.9	10.2	10.8	1.0	4.5	3.5	-1.23		2.14	
Bol009609	AT4G23810	WRKY53	52.7	149.1	78.1	7.3	54.3	66.3	1.51		2.89	
Bol042130	AT4G23810	WRKY53	9.3	7.9	6.9	0.2	3.4	4.7			4.15	
Bol005121	AT2G40750	WRKY54	78.4	55.0	48.4	17.3	49.8	75.1			1.52	
Bol006887	AT2G40750	WRKY54	2.5	0.6	0.3	0.6	0.4	0.4	-2.83			
Bol002547	-	WRKY55	0.3	-	1.4	0.1	0.1	0.2		1.44		
Bol000854	AT5G01900	WRKY62	0.4	0.4	2.3	0.8	1.0	2.6		2.64		
Bol005975	AT5G01900	WRKY62	19.1	7.1	9.0	2.2	1.9	1.1	-1.43			
Bol004954	AT3G58710	WRKY69	3.6	6.0	4.6	4.1	8.8	6.5			1.98	
Bol044275	AT3G56400	WRKY70	34.1	8.1	5.6	2.8	8.3	15.5	-2.72		1.58	
Bol002548	AT3G56400	WRKY70	79.7	41.8	45.1	8.7	40.3	35.7			2.22	
Bol001387	-	WRKY74	4.9	6.8	3.2	5.9	4.6	5.0		-1.93		
Bol034214	AT5G13080	WRKY75	4.4	2.3	15.2	0.8	2.7	7.7		2.76		1.52

Continued Transcriptome changes for genes involved in antioxidation, Ca^{2+} signalling and WRKY transcription factors.

Rice (Oryza sativa)

Gene ID	*Arabidopsis homologue*	Description	RPKM value			Log2(Fold change)	
			Os0	Os6	Os12	Os6-0	Os12-6
LOC_Os02g02400	-	Catalase isozyme A	238.1	730.6	289.6	1.62	-1.33
LOC_Os07g48060	-	Cationic peroxidase 1	0.5	10.7	4.0	4.15	-1.42
LOC_Os01g73170	AT1G71695	Cationic peroxidase SPC4 (Precursor)	48.7	163.0	134.5	1.75	
LOC_Os01g73200	AT1G71695	Cationic peroxidase SPC4 (Precursor)	0.3	118.3	130.9	8.25	
LOC_Os04g59190	-	Cationic peroxidase SPC4 (Precursor)	0.3	15.9	3.6	5.32	-2.14
LOC_Os04g59210	-	Cationic peroxidase SPC4 (Precursor)	0.0	0.9	0.2	3.69	
LOC_Os01g22230	-	Peroxidase 1 (Precursor)	1.0	5.6	3.8	2.45	
LOC_Os01g22352	-	Peroxidase 1 (Precursor)	2.5	13.4	13.3	2.41	
LOC_Os01g22370	-	Peroxidase 1 (Precursor)	12.8	34.1	41.4	1.43	
LOC_Os01g19020	-	Peroxidase 2 (Precursor)	0.1	10.1	1.0	5.86	-3.25
LOC_Os07g48010	-	Peroxidase 2 (Precursor)	9.1	119.9	78.8	3.71	
LOC_Os07g48020	-	Peroxidase 2 (Precursor)	44.7	303.8	482.5	2.77	
LOC_Os06g46799	AT1G05260	Peroxidase 3 (Precursor)	1.1	11.3	9.5	3.34	
LOC_Os04g55740	-	Peroxidase 4	0.1	6.4	0.7	5.15	-3.23
LOC_Os03g02920	-	Peroxidase 5	5.0	15.6	4.6	1.64	-1.75
LOC_Os04g59150	-	Peroxidase 12	14.5	257.0	271.4	4.16	
LOC_Os10g02040	AT2G38380	Peroxidase 15	0.5	21.1	18.8	5.14	
LOC_Os06g48030	AT3G49960	Peroxidase 16 (Precursor)	1.1	23.0	22.3	4.30	
LOC_Os09g29490	AT2G22420	Peroxidase 17 (Precursor)	36.1	235.0	373.8	2.86	
LOC_Os01g16450	AT2G39040	Peroxidase 24 (Precursor)	0.6	8.0	0.7	3.69	-3.44
LOC_Os06g32990	-	Peroxidase 36 (Precursor)	0.8	103.5	174.4	6.84	
LOC_Os08g02110	AT4G33420	Peroxidase 47 (Precursor)	10.1	19.9	40.4		1.02
LOC_Os06g35520	-	Peroxidase 52 (Precursor)	0.2	17.6	4.9	5.94	-1.83
LOC_Os02g14430	-	Peroxidase 70 (Precursor)	2.9	256.4	161.6	6.43	
LOC_Os03g13210	-	Peroxidase N (Precursor)	5.2	96.0	42.9	4.20	-1.16
LOC_Os10g02070	AT5G19890	Peroxidase N (Precursor)	0.2	19.6	10.0	5.90	

Supplenmentary Table 5 Significantly pathways (*Q* < 0.05) in resistant and susceptible groups of *Brassica oleracea* during 0 to 12 hpi by *Sclerotinia sclerotiorum*

ID	Pathway	Genes in genome	R6 vs. R0			S6 vs. S0			R12 vs. R6			S12 vs. S6		
			Nr. DEGs	Q value	Up ratio*	Nr. DEGs	Q value	Up ratio	Nr. DEGs	Q value	Up ratio	Nr. DEGs	Q value	Up ratio
ko04626	Plant-pathogen interaction	1643	247	2.0E-03	32.4%	178	5.0E-02	69.1%						
ko04075	Plant hormone signal transduction	1634	261	4.0E-05	34.9%	191	2.0E-03	56.5%						
ko01100	Metabolic pathways	5034	726	4.0E-06	33.5%	586	5.0E-10	37.5%	242	1.0E-06	56.2%	432	3.0E-02	34.3%
ko00500	Starch and sucrose metabolism	651	125	4.0E-06	24.8%	93	1.0E-04	36.6%						
ko01110	Biosynthesis of secondary metabolites	2791	462	7.0E-11	30.5%	388	2.0E-16	34.0%	140	2.0E-04	67.1%			
ko00941	Flavonoid biosynthesis	297	70	1.0E-06	18.6%	56	2.0E-06	25.0%						
ko00940	Phenylpropanoid biosynthesis	490	112	2.0E-09	25.0%	93	4.0E-10	33.3%	35	1.0E-03	57.1%			
ko00944	Flavone and flavonol biosynthesis	93	20	3.0E-02	55.0%	21	7.0E-04	57.1%						
ko00591	Linoleic acid metabolism	38	11	2.0E-02	27.3%	12	7.0E-04	25.0%						
ko00053	Ascorbate and aldarate metabolism	157	31	2.0E-02	19.4%	24	4.0E-02	29.2%						
ko00966	Glucosinolate biosynthesis	105	26	2.0E-03	15.4%	31	6.0E-08	19.4%	16	2.0E-05	100.0%			
ko00945	Stilbenoid, diarylheptanoid and gingerol biosynthesis	370	74	1.0E-04	41.9%	62	3.0E-05	46.8%						
ko00592	alpha-Linolenic acid metabolism	144	37	9.0E-05	32.4%	31	6.0E-05	35.5%						
ko00360	Phenylalanine metabolism	245	59	4.0E-06	28.8%	45	6.0E-05	31.1%	21	2.0E-03	76.2%			
ko00906	Carotenoid biosynthesis	213	50	5.0E-05	14.0%	45	2.0E-06	22.2%				29	3.0E-02	27.6%
ko00400	Phenylalanine, tyrosine and tryptophan biosynthesis	93	22	1.0E-02	22.7%	20	2.0E-03	10.0%						

ko00052	Galactose metabolism	125	27	1.0E-02	63.0%	21	2.0E-02	66.7%	11	4.0E-02	90.9%	20	3.0E-02	30.0%
ko00903	Limonene and pinene degradation	279	49	3.0E-02	55.1%	42	6.0E-03	57.1%						
ko04712	Circadian rhythm - plant	302	83	7.0E-11	48.2%	77	4.0E-15	55.8%	37	2.0E-09	59.5%	59	7.0E-10	30.5%
ko00910	Nitrogen metabolism	116	33	3.0E-05	30.3%	22	5.0E-03	22.7%	13	2.0E-03	84.6%			
ko00380	Tryptophan metabolism	207	47	2.0E-04	29.8%	46	3.0E-07	30.4%	29	8.0E-09	93.1%			
ko00604	Glycosphingolipid biosynthesis - ganglio series	37	10	4.0E-02	60.0%	10	8.0E-03	60.0%						
ko00460	Cyanoamino acid metabolism	170	43	3.0E-05	18.6%	38	3.0E-06	26.3%	17	1.0E-03	64.7%			
ko00280	Valine, leucine and isoleucine degradation	95	21	2.0E-02	61.9%				9	5.0E-02	88.9%			
ko00920	Sulfur metabolism	85				20	6.0E-04	20.0%	9	3.0E-02	100.0%			
ko00196	Photosynthesis - antenna proteins	40							14	1.9E-09	0.0%	25	1.2E-16	4.0%
ko00300	Lysine biosynthesis	46							6	4.1E-02	83.3%			
ko00630	Glyoxylate and dicarboxylate metabolism	97							9	4.9E-02	44.0%			
ko00620	Pyruvate metabolism	195							16	1.5E-02	87.5%			
ko00195	Photosynthesis	134							25	9.4E-10	4.0%			
ko00562	Inositol phosphate metabolism	167	31	4.0E-02	54.8%									
ko00350	Tyrosine metabolism	98	27	3.0E-04	51.9%									
ko00950	Isoquinoline alkaloid biosynthesis	52	13	3.0E-02	46.2%									
ko00960	Tropane, piperidine and pyridine alkaloid biosynthesis	92	21	2.0E-02	38.1%									
ko00130	Ubiquinone and other terpenoid-quinone biosynthesis	99	24	4.0E-03	33.3%									

ko00260	Glycine, serine and threonine metabolism	112	27	2.0E-03	25.9%			
ko00520	Amino sugar and nucleotide sugar metabolism	286	50	3.0E-02	24.0%			
ko00270	Cysteine and methionine metabolism	214	39	3.0E-02	23.1%			
ko00040	Pentose and glucuronate interconversions	359	69	7.0E-04	14.5%			
ko03010	Ribosome	709	122	5.0E-04	7.4%			
ko00531	Glycosaminoglycan degradation	43				10	2.0E-02	60.0%
ko02010	ABC transporters	147				26	5.0E-03	50.0%
ko01040	Biosynthesis of unsaturated fatty acids	107				18	4.0E-02	33.3%
ko00902	Monoterpenoid biosynthesis	15				5	4.0E-02	20.0%

* The 'Up ratio' represents the percentage of up-regulated genes in the total altered genes envolved in a certain pathway

Supplementary Figure 1 Expression changes as estimated by RNA-Seq (black bars) and RT-PCR (grey bars) for 46 genes. Errors bars represent the standard deviation of three biological replicates of RT-PCR. The last 19 genes were normalized with *Actin3* and *actin7*, while the others were normalized with *Actin3*.

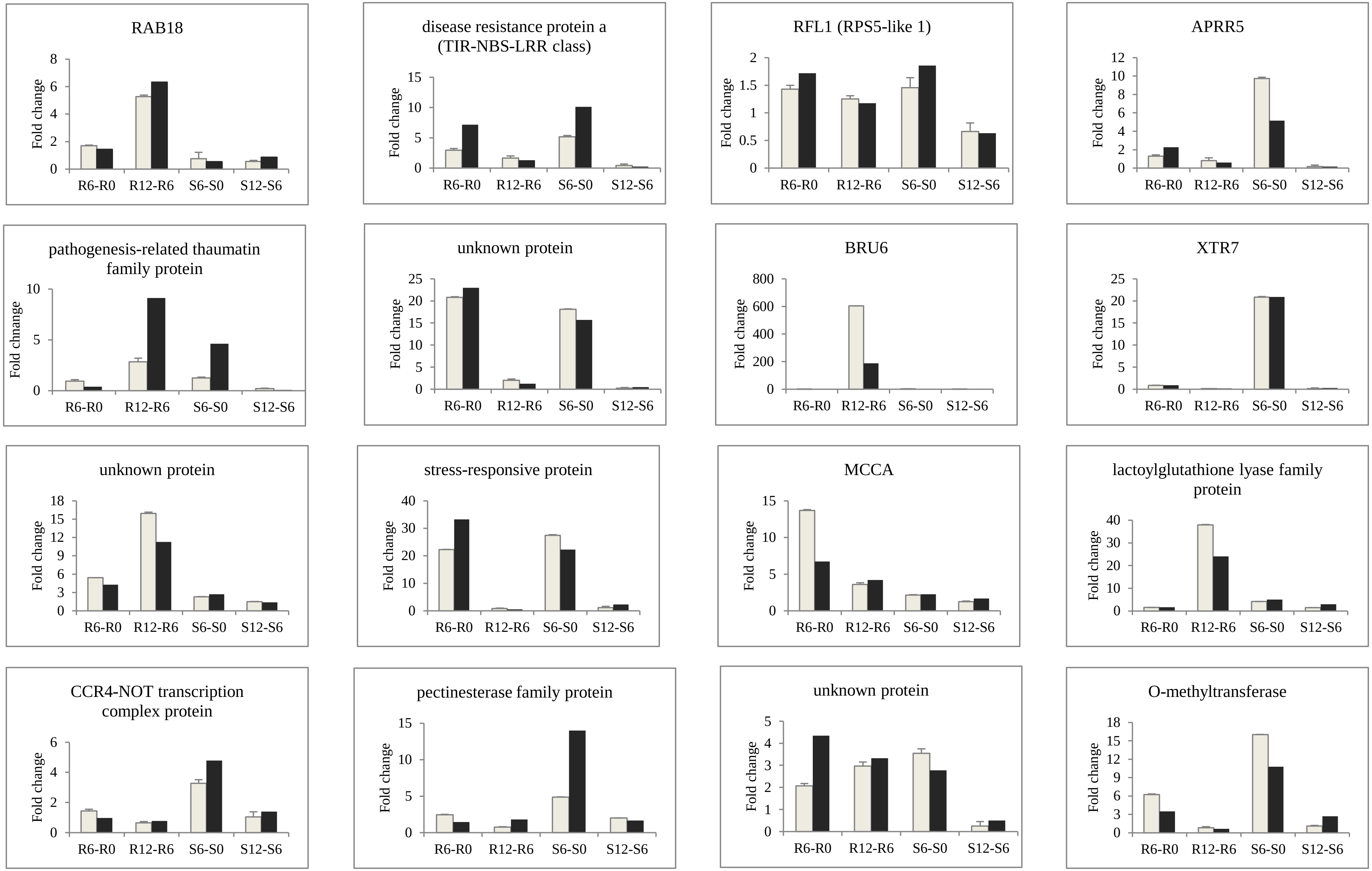

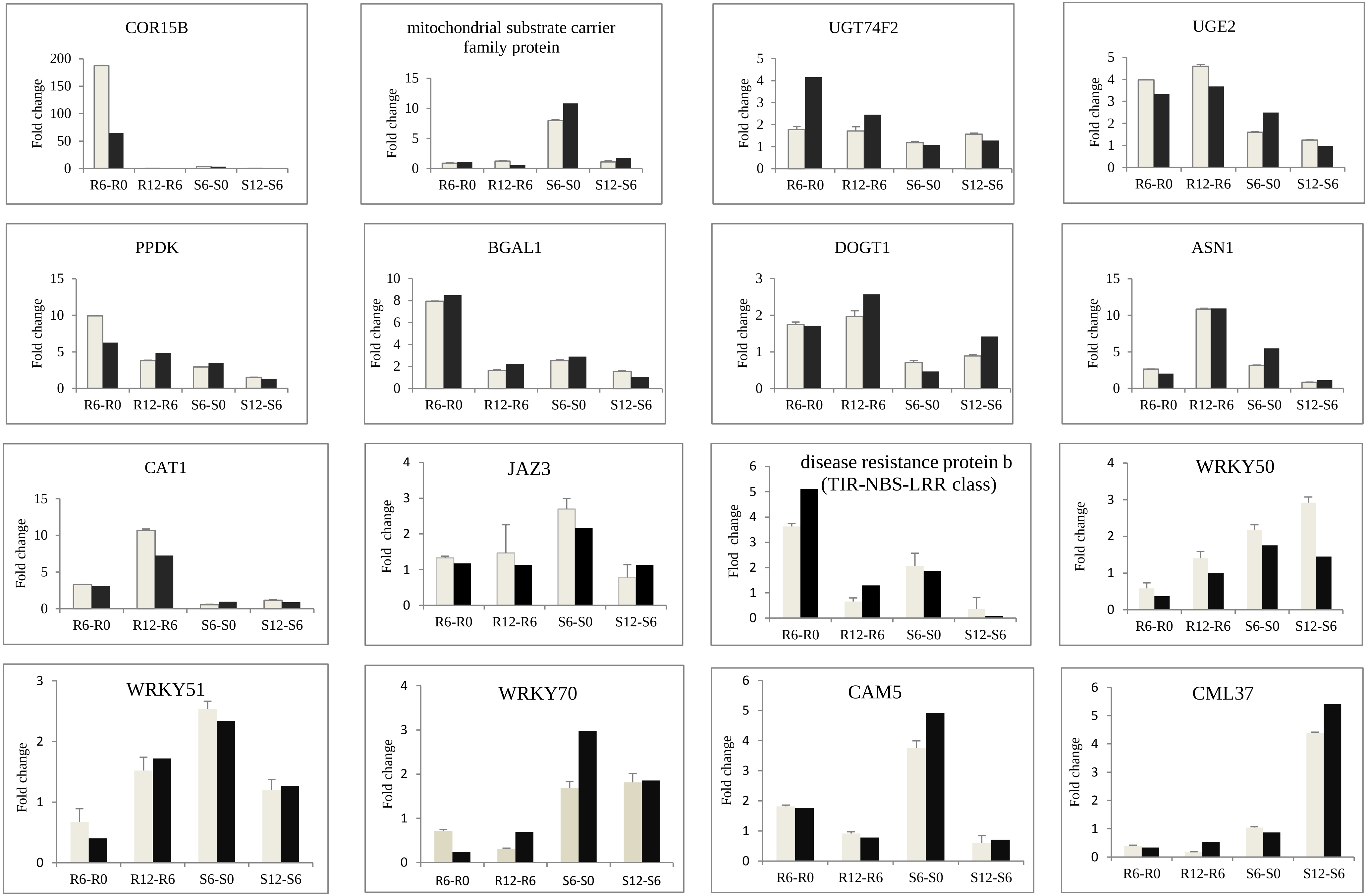
COR15B
mitochondrial substrate carrier family protein
UGT74F2
UGE2
PPDK
BGAL1
DOGT1
ASN1
CAT1
JAZ3
disease resistance protein b (TIR-NBS-LRR class)
WRKY50
WRKY51
WRKY70
CAM5
CML37
Fold change
Flod change
R6-R0
R12-R6
S6-S0
S12-S6

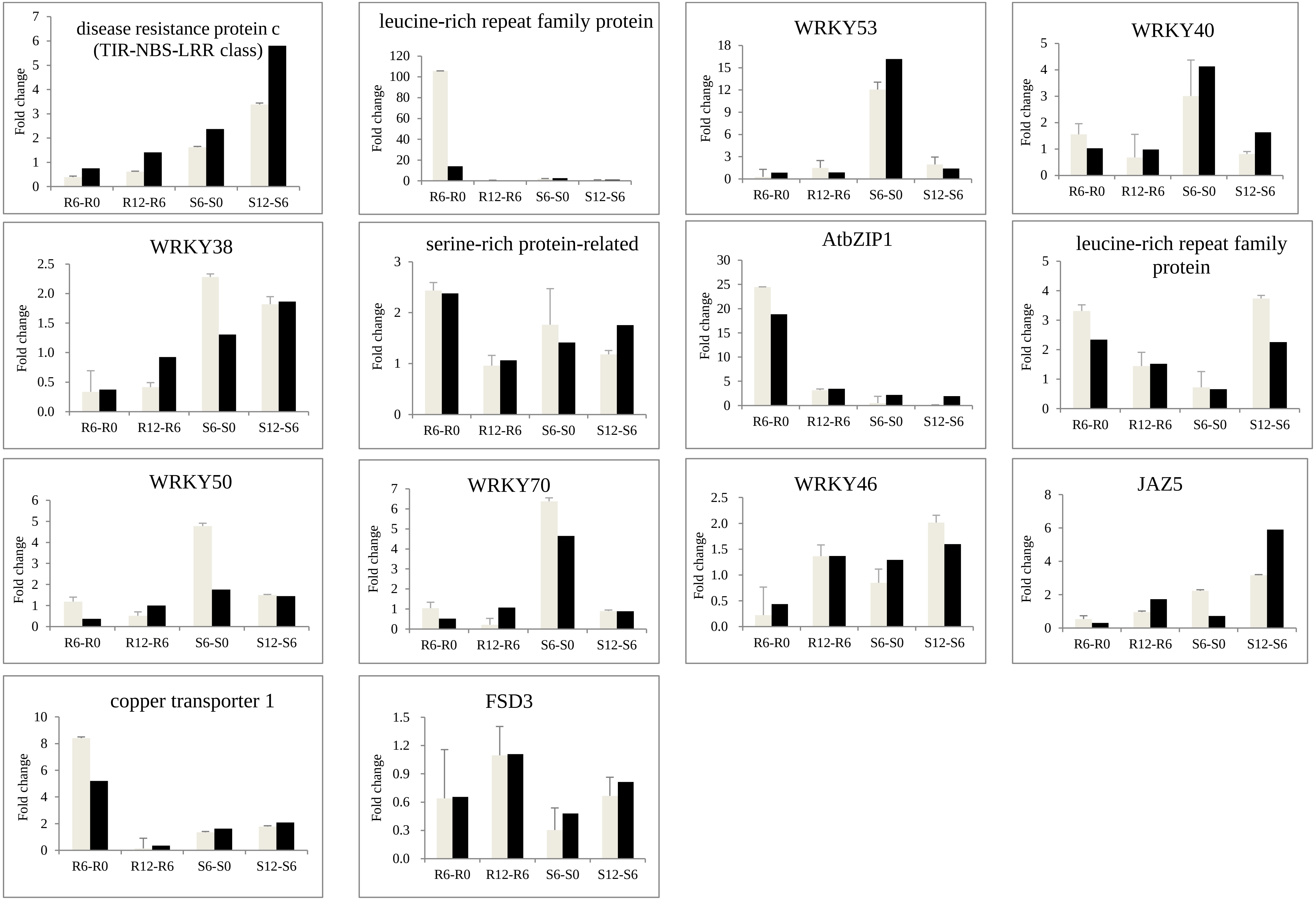

disease resistance protein c (TIR-NBS-LRR class)
leucine-rich repeat family protein
WRKY53
WRKY40
WRKY38
serine-rich protein-related
AtbZIP1
leucine-rich repeat family protein
WRKY50
WRKY70
WRKY46
JAZ5
copper transporter 1
FSD3
Fold change
R6-R0
R12-R6
S6-S0
S12-S6

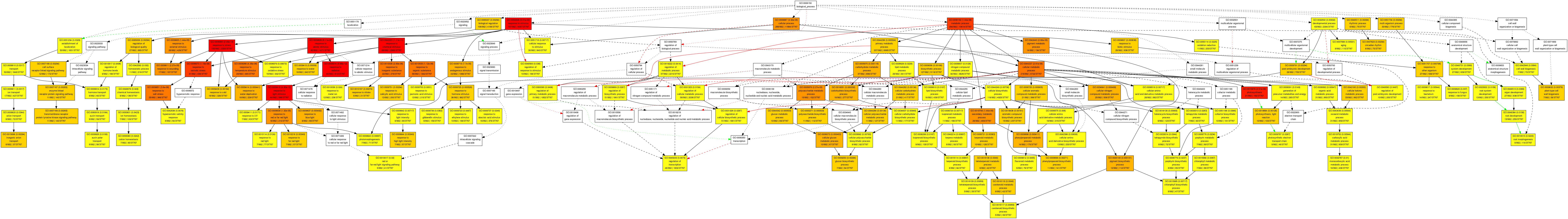

Supplement: Supplementary Information [file srep33706-s1.pdf]
